# Production of kidney organoids arranged around single ureteric bud trees, and containing endogenous blood vessels, solely from embryonic stem cells

**DOI:** 10.1038/s41598-022-16768-1

**Published:** 2022-07-22

**Authors:** Anwar A. Palakkan, Julia Tarnick, Martin Waterfall, May Sallam, Fokion Glykofrydis, Mona Elhendawi, Jamie A. Davies

**Affiliations:** 1 Deanery of Biomedical Science, University of Edinburgh, Hugh Robson Building, George Square, Edinburgh, EH8 9XD UK; 2 Institute of Immunology & Infection Research, Ashworth Laboratories, School of Biological Sciences, University of Edinburgh, King’s Buildings, Edinburgh, EH9 3JD UK; 3 Human Anatomy and Embryology Department, Faculty of Medicine, Mansoura University, Mansoura, Egypt; 4 Clinical Pathology Department, Faculty of Medicine, Mansoura University, Mansoura, Egypt; 5 UK Centre for Mammalian Synthetic Biology, University of Edinburgh, CH Waddington Building, King’s Buildings, Mayfield Road, Edinburgh, EH9 3JD UK; 6 Present Address: Immunology and Stem Cell Biology, Aravind Medical Research Foundation, Madurai, 625020 India

**Keywords:** Biological techniques, Cell biology, Developmental biology, Stem cells, Anatomy, Nephrology, Urology

## Abstract

There is intense worldwide effort in generating kidney organoids from pluripotent stem cells, for research, for disease modelling and, perhaps, for making transplantable organs. Organoids generated from pluripotent stem cells (PSC) possess accurate micro-anatomy, but they lack higher-organization. This is a problem, especially for transplantation, as such organoids will not be able to perform their physiological functions. In this study, we develop a method for generating murine kidney organoids with improved higher-order structure, through stages using chimaeras of ex-fetu and PSC-derived cells to a system that works entirely from embryonic stem cells. These organoids have nephrons organised around a single ureteric bud tree and also make vessels, with the endothelial network approaching podocytes.

## Introduction

Organoids generated from stem cells are valuable tools for modelling diseases, for testing drugs, and for producing transplantable replacement organs. Protocols have been developed for generating kidney organoids from mouse ex-fetu renogenic stem cells^1–3^ and from mouse and human pluripotent stem cells (PSC)^4–7^. Organoids contain more than one cell type, a fact that enables them to model physiology more accurately than single cell-type cultures. They can also replicate pathogenesis involving interactions between different cell types^8^. Though protocols for generating kidney organoids from stem cells are well established, those from PSC lack the anatomical higher order of a real kidney, their nephrons not being connected to a single urine collecting system^9^.

Normal kidneys are patterned by the interactions between the tips of a sequentially branching structure, the ureteric bud (UB), and the surrounding metanephric mesenchyme^10^ that contains nephron progenitor (NP) and stromal progenitor (SP) cells. Interactions between these populations are highly regulated^11^ and give rise to an arrangement in which excretory nephrons, which develop from NP, filter blood at their glomerular ends and recover solutes through their tubular portions, connect to the outer branches of a single, coherent collecting duct tree, which develops from UB. Traditionally, stromal cells were considered to support kidney development by secreting extracellular matrix and growth factors. However, more specific roles of stromal cells in the regulation of both UB branching and nephron formation are now recognized^12^, and stromal ablation studies cause severe structural deformities during mouse kidney development^13^.

Organoids produced either by re-aggregating disaggregated mouse ex-fetu renogenic stem cells^3^ or by the differentiation of PSC^4,5,14,15^ have realistic anatomy at the micro-scale (nephrons etc.) but lack organotypic higher-order. This problem was addressed some years ago by our lab for organoids made from mouse ex-fetu cells, by a serial aggregation system that provided the UB / collecting duct progenitor cells in the form of a single, coherent epithelial tubule^1^. These organoids were further improved by asymmetric application of BMP4 to create a ureter-type structure from one end of the collecting duct tree^2^. This single-tree-with-ureter arrangement has not yet been achieved for organoids made entirely from PSC. The closest approaches so far have been from the Nishinakamura laboratory^6,16^, and Zeng and colleagues'^17^. In their first publication on this topic, the Nishinakamura laboratory produced mouse ESC-derived ureteric bud branches, and mouse ESC-derived nephron progenitors. Then, by mixing one mESC-derived ureteric bud branch with mESC-derived nephron progenitors and with ex-fetu mouse stromal cells, they produced organoids based on a single collecting duct tree. It was an important step forward, but they still had to use ex-fetu mouse materials for stromal progenitors, which were necessary for the formation of the organ, and their kidneys featured little vasculature. In their second, very recent publication^16^ that appeared during the revision phase of our current report, the Nishinakamura lab developed a differentiation protocol to make mouse dorsal stromal progenitors from mouse ESC. They showed that these could substitute for ex-fetu stromal progenitors by combining them with mESC-derived ureteric bud and mESC-derived nephron progenitors to make kidney organoids based on a single ureteric bud/ collecting duct tree. There was no information on extent of vessel formation in culture, though there was evidence of vessel formation when the organoids were transplanted to a host mouse. Zeng and colleagues' lab made ureteric bud organoids from ex-fetu mouse ureteric tip cells expanded in 3D culture and added them to ex-fetu mouse metanephric mesenchyme expanded in culture in a way believed to produce cultures consisting only of nephron progenitor cells: the result was formation of a kidney organoid with nephrons arranged around a single collecting duct tree, albeit with no ureter and with the degree of vascular development unmeasured. The apparent conflict between the observations of the Nishinakamura laboratory that stromal cells are required, and the Zeng one that they are not, may simply reflect the different way that 'nephron progenitor cells' were made (the same phrase 'nephron progenitor cell' in the two papers probably does not mean precisely the same thing).

This report describes another method for producing mouse kidney organoids centred on a single ureteric bud/ collecting duct system, from all-mESC-derived cells with no need, in the final method we describe, for any ex-fetu cells. It demonstrates that nephrons in these organoids are arranged on a single ureteric bud tree, which can be differentiated locally to ureter character, that the organoids also have a network of endothelial cells, and that a branch of the ureteric bud tree can be induced by a local source of BMP4 to differentiate to ureter-type urothelium rather than collecting duct. This is a step forward in the anatomical realism of kidney organoids developed from PSC and a complement to other methods being developed.

## Results

For this study, kidney organoids were generated by aggregating a ureteric bud with NP and SP; all of them where either derived from mouse embryonic stem cells (mESC) or isolated from embryonic kidney, according to the experiment being done. We used three types of organoids, moving in stages from all-ex-fetu (essentially verification of previously-published data as a foundation for what follows) to all-mESC-derived (which is entirely new). We refer to them as follows: ‘ex-fetu organoids’, in which all the above cell types were derived from ex fetu renogenic stem cells isolated from the developing mouse embryonic kidney; ‘chimeric organoids’, in which some cell types were mESC-derived cells and others ex-fetu; ‘all-mESC organoids’, in which all cells came from mESC.

This Results section is divided into three main parts. First, we describe production and characterization of engineered ureteric bud (eUB), induced nephron progenitors (iNP) and induced stromal progenitors (iSP) from mESC. Second, we verify the developmental potential of these cell types by making chimeric organoids, with the other cell types being isolated directly from mouse embryos. Third, we describe the production and characterization of all-mESC-derived organoids.

### Production and characterization of mESC derived ureteric Bud (eUB)

**Figure 1:**
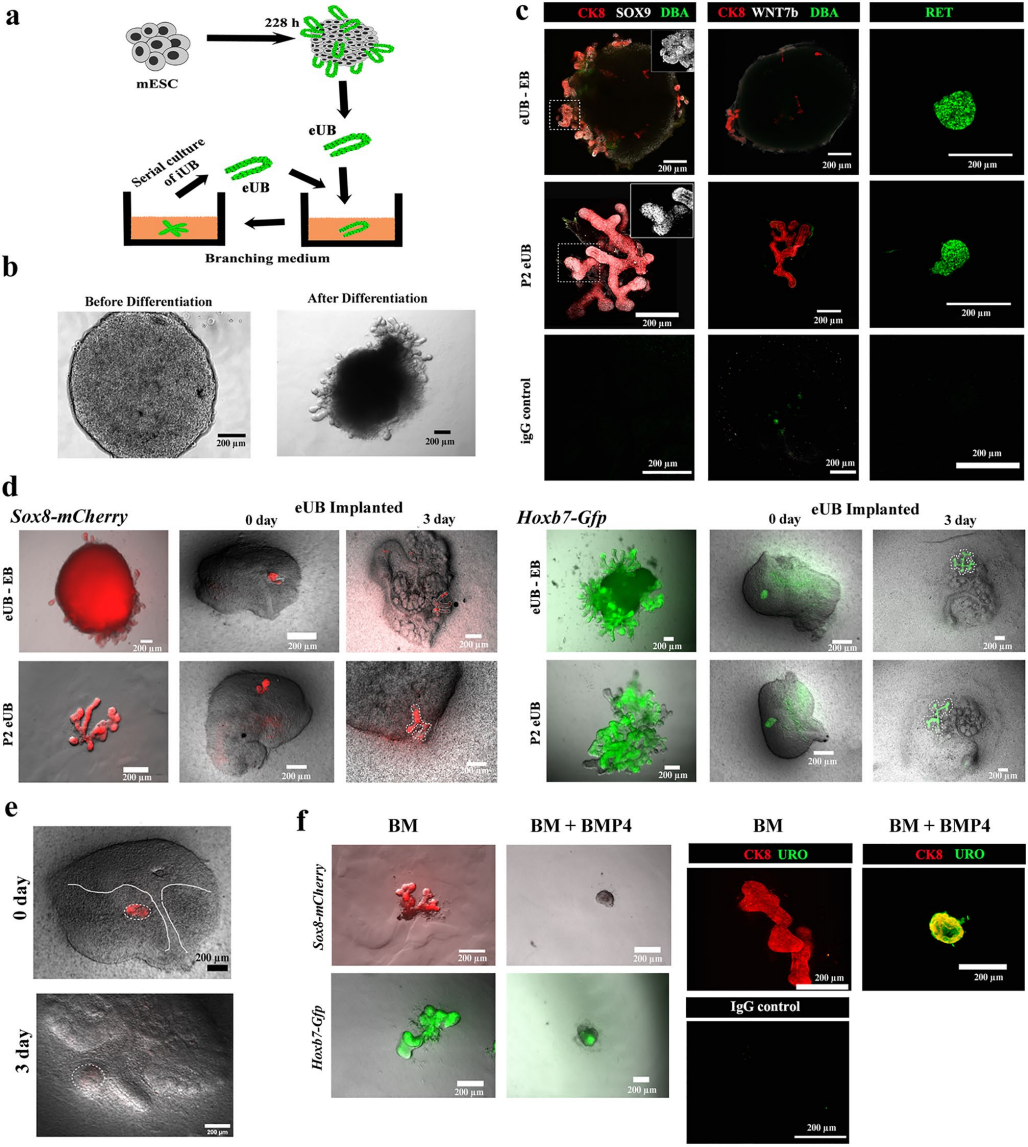
Differentiation of mESC to ureteric bud cells (eUB) (**a**) Schematic diagram showing the isolation and culture of eUBs in branching medium. (**b**) Representative image of embryoid bodies before (48 h) and after differentiation (228 h). Scale bar, 200 µm (**c**) Immunostaining for tip (SOX9, RET) and trunk markers (DBA, WNT7b) in outgrowths from differentiated embryoid bodies (eUB-EB) and passage 2 eUBs (P2 eUB). Cytokeratin 8 (CK8) is a general ureteric bud marker. Inset figure shows the zoomed view of the SOX9 channel in the marked area (dotted line). *n* = 5/5. (**d**) Fluorescence images of eUBs (derived from *Sox8-mCherry* and *Hoxb7-Gfp* lines) implanted in metanephric mesenchyme (from E11.5 kidney), showing branching. eUBs isolated from differentiated embryoid bodies (eUB-EB) and passage 2 eUBs (P2 eUB) were used for implantation studies. Images of kidney at Day 0 and Day 3 after implantation is shown. *n* = 3/3. (**e**) Fluorescence images of eUBs (derived from the *Sox8-mCherry* line) implanted in ureteric mesenchyme (from a E11.5 kidney) showing absence of branching at day 3. *n* = 0/3. (**f**) Branching morphogenesis of eUBs in branching medium (BM) with BMP4 (BM + BMP4) or without BMP4 (BM): BMP4 inhibits branching but promotes expression of uroplakin: *n* = 3/3 for BM; *n* = 5/5 for BM + BMP4. Scale bars, 200 µm.

We produced mESC-derived eUBs using a protocol established in our lab^18,19^ by modifying a previous method^6^ (details are provided in method section and Supplementary Fig. 1a). The process of eUB differentiation from mESC, and in vitro branching of the product, is depicted diagrammatically in Fig. 1a. By the end of the differentiation process (approximately 228 h), the cell cultures had developed numerous budding structures (Fig. 1b). We follow our established practice of referring these mESC-derived buds as engineered ureteric buds, eUBs. When isolated from their parent culture using a sharp needle and placed in a 3D gel rich in growth factors (branching medium), eUBs underwent branching morphogenesis [*n* = 18/20, 90% underwent branching; 95% confidence internal (CI): 74% to 100%]. We were able to propagate buds from these branched structures serially for many passages but, for this study, their continued health (ability to grow and branch) was evaluated systematically only up to passage 4, counting the original culture as 0 [*n* = 18/18, 100% healthy; CI: 97% to 100%]. For subsequent experiments, either freshly isolated or passage-2 eUBs were used. Throughout the culture period, the branching structures made from *Hoxb7-Gfp* mESC expressed GFP, and those made from *Sox8-mCherry* mESC expressed mCherry (Supplementary Fig. 1b) [*n* = 6/6, 100% expressed; CI: 92% to 100%].

In an embryonic kidney, the UB has two distinct zones, an actively branching tip and a differentiated trunk part that rarely branches. Our eUBs showed the expression of the tip markers, SOX9^20,21^ and RET^21–23^ [*n* = 5/5, 100% expressed; CI: 90% to 100%]. They expressed neither the trunk marker WNT7b^21^ nor bound labelled Dolichos biflorus agglutinin (DBA) [*n* = 0/5, 0% expressed; CI: 0% to 10%], also characteristic of trunk^18,24^, either in their original culture or when passaged and cultured in branching medium (Fig. 1c). These results show that our eUBs were more like UB tip than trunk.

Ex-fetu isolated UBs display plasticity, developing into either branched collecting ducts or unbranched urothelium in response to their environment. Our previous studies have demonstrated this plasticity of eUBs to form a branched collecting duct system when surrounded by ex-fetu metanephric mesenchyme, and to form an unbranched ureter-type urothelial differentiation when surrounded by ureteric mesenchyme^18,19^. To gain further insight on the plasticity and tissue identity of eUBs, we decided to investigate the expression of *Hoxb7* and *Sox8* using reporting mESC lines. The *Hoxb7*gene is expressed constitutively by all UB cells, irrespective of being tip or ureter. On the other hand, *Sox8* is involved in UB outgrowth^20^ and is expressed in the tips, but is completely absent in ureter regions^25^. In agreement with the findings from Sallam and colleagues^18^, our eUBs branched to form a tree-like morphology when implanted into ex-fetu metanephric mesenchyme (Fig. 1d) [*n* = 3/3, 100% branched; CI: 83% to 100%]. Expression of *Sox8-mCherry* was observed throughout the branching structure but was brighter at the tips. When *Sox8-mCherry* eUBs were implanted into ex-fetu ureteric mesenchyme, they remained unbranched and lost mCherry expression (Fig. 1e) [*n* = 0/3, 0% positive; CI: 0% to 17%]. It is believed that ureteric buds respond to morphogens in the surrounding mesenchyme: BMP4 is one such morphogen and has an important role in UB tip/ureter distinction^26,27^. We conducted simple experiments to test if eUBs can be differentiated towards ureteric lineage in in vitro just by providing signalling molecules, without a ureteric mesenchyme. In the presence of BMP4, our eUBs underwent little or no branching, but instead became spherical in shape, lost SOX8 expression and expressed the urothelium marker, uroplakin [*n* = 5/5, 100% expressed; CI: 90% to 100%]. In controls (branching medium without BMP4), eUBs branched, showed SOX8 expression and were negative for uroplakin expression (Fig. 1f) [*n* = 3/3, 100% expressed SOX8; CI: 83% to 100%]. To the authors' knowledge this is the first report showing that eUBs can respond to BMP4 under in vitro conditions. The above results clearly show that, though eUBs generated were more like UB tip, they were able to respond to the surrounding mesenchymal signals and can become either collecting duct or ureter.

To test whether there might be significant differences between eUBs and ex-fetu UBs, we performed RNA sequencing. We used E11.5 ex-fetu kidneys as a comparison, as they include ureteric buds that have begun to branch but not yet differentiated into mature collecting duct. The 50 most differentially expressed genes are shown in Supplementary Fig. L1. Clearly the difference between ES-derived eUB cells and ex-fetu UB cells dominates over variation between samples of the same cell type. We ran a Geneontology/ Panther analysis of biological processes associated with these most differential genes. The analysis highlighted the processes of morphogenesis, locomotion and signalling as being raised in the eUBs. The most probable reason for this is that eUBs are more tip-like than whole ex-fetu UB. The appearance of *Calb1*, stronger in stalk than tip, in the list of genes less expressed in eUBs than in ex-fetu UBs, supports this interpretation.

### Production and characterization of mESC-derived nephron- and stromal-progenitors from mESCs

During embryogenesis, intermediate mesoderm gives rise to metanephric mesenchyme in which NP and SP coexist^28^. NP are largely believed to be derived from intermediate mesoderm. The origin of SP is debatable, and some reports suggest a paraxial mesoderm origin^29^. Currently available stem cell differentiation protocols are formulated for generating NP from intermediate mesoderm cells, though a small population of stromal like cells are also observed in these differentiation protocols^14,30^. Activin, WNT and BMP signalling are commonly used to control differentiation^31^, and can also induce mesoderm progenitor cells at a different concentration^32^. We were interested in having both mESC-derived nephron and stromal progenitors (iNP and iSP) at almost equal proportions in the same culture, and for this purpose we modified an existing method^6,14^ by adjusting Activin A and BMP4 concentration during the initial steps (Supplementary Fig. 1a, Supplementary Fig. 1c).

**Figure 2:**
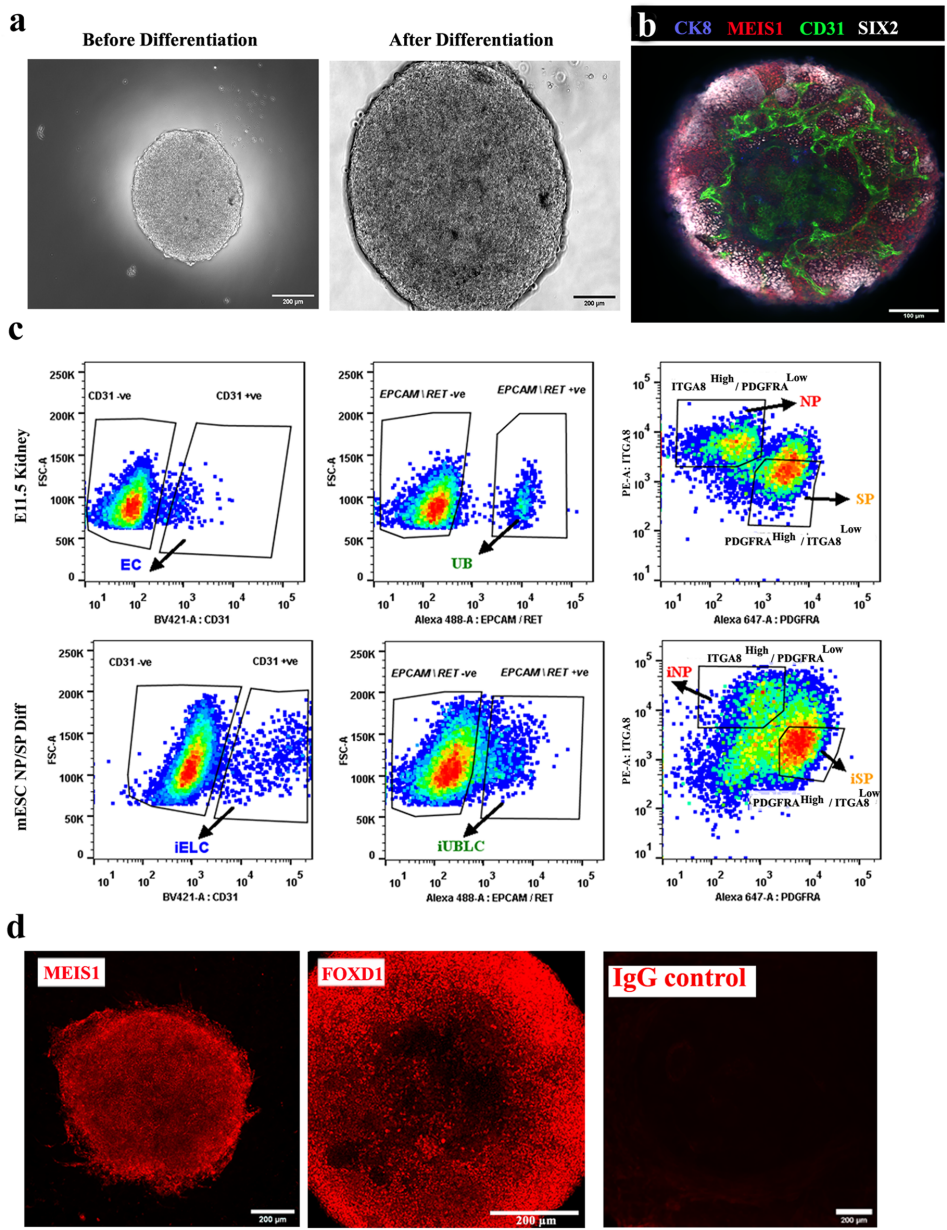
Differentiation of mESC to nephron and stromal progenitors (iNP, iSP). (**a**) Representative images of embryoid bodies before (48 h) and after differentiation (228 h). Scale bar, 200 µm. (**b**) Immunostaining for endothelial (CD31), ureteric bud (CK8), nephron progenitor (SIX2) and stromal progenitors (MEIS1) in differentiated embryoid bodies. *n* = 5/5. Scale bar, 100 µm. (**c**) Representative flow cytometry data of E11.5 kidney and differentiated embryoid bodies (mESC NP/SP Diff) showing the ITGA8^high^/PDGFRA^low^ gate for NP/iNP cells and the INTGA8^low^ / PDGFRA^high^ gate for SP/iSP) cells. Ureteric bud (EpCAM/RET + ve) and endothelial cells (CD31) were negatively gated before sorting NP/iNP and SP/iSP cells. Abbreviations: EC: endothelial cells, UB: ureteric bud, iELC: induced endothelial like cells, eUBLC: engineered ureteric bud like cells. (**d**) Immunostaining for MEIS1 and FOXD1 in sorted iSP cells. *n* = 3/3. Scale bar, 200 µm.

By the end of the differentiation process (approximately 228 h), embryoid bodies (EBs) had grown extensively, acquired a granular appearance (Fig. 2a), and had mESC-derived endothelial cells (CD31-positive cells), ureteric bud cells (CK8 +), nephron progenitors (SIX2 +) and stromal progenitors (MEIS1 +) (Fig. 2b) [*n* = 5/5, 100% had these features; CI: 90% to 100%]. We refer these mESC-derived nephron and stromal progenitors as induced nephron progenitors (iNP) and induced stromal progenitors (iSP) respectively.

Our initial intention had been to obtain pure populations of iNP and iSP, or of their ex-fetu equivalents by cell sorting, based on the expression of ITGA8 and PDGFRA^6^. In this study, ITGA8^High^/PDGFRA^Low^ cells were considered to be nephron progenitors (NP/iNP), as reported before ^6,33,34^. PDGFRA expressing cells have been considered stromal progenitors in previous studies^6,35^. Considering the diversity of stromal cells^36,37^, we selected only the densest sub-population of PDGFRA^+ve^ cells (ITGA8^Low^/PDGFRA^High^) to reduce variability between cultures, and provisionally called them stromal progenitors (SP/iSP) (Fig. 2c).

**Figure 3:**
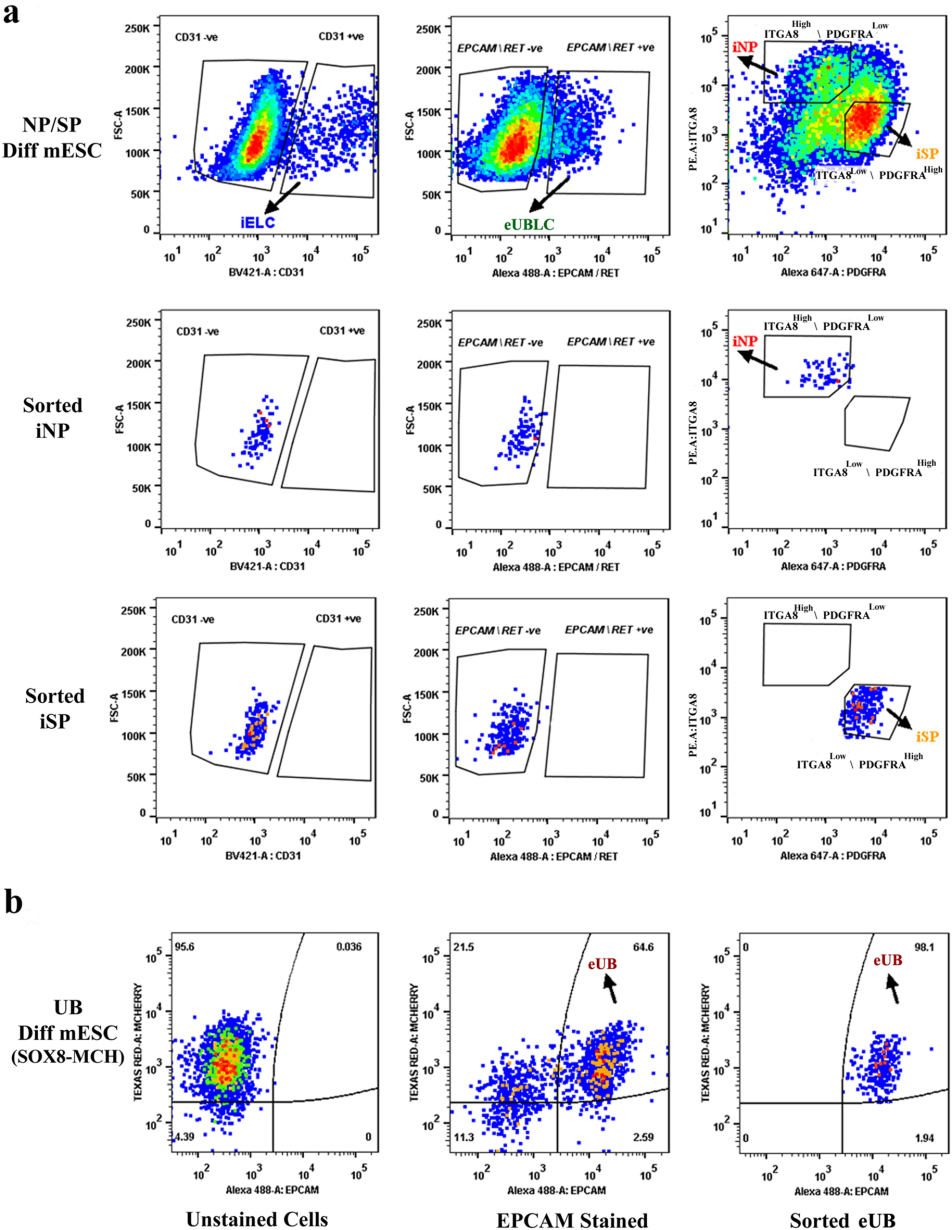
Purity of sorted progenitors from differentiated mESC cells. The top 3 rows refer to embryoid bodies differentiated towards iNP/ iSP cells and the bottom row refers to those differentiated towards eUB. (**a**) The top row shows profiles of unsorted iNP/ iSP-differentiated mESC with respect to the endothelial marker CD31, the ureteric bud markers EpCAM/RET, and the sorting profile based on ITGA8 and PDGFRA. The other rows show post-sorting populations of iNP and iSP: it can be seen that they are free from endothelial (iELC: induced endothelial-like cells) and ureteric bud (eUBLC: engineered ureteric bud like cells) contamination. (**b**) shows the staining profile of eUB-differentiated embryoid bodies with respect to SOX8-mCherry and EpCAM, the sorting profile, and the purity of the resultant sorted cells.

To avoid potential contamination by UB (ex-fetu or mESC-derived), we sought a sorting (exclusion) antibody for these cells. EPCAM is an epithelial-specific adhesion molecule and had been reported to be expressed only in the UB at these stages of development^38^. We confirmed this restricted expression in E12.5 kidney and differentiated EBs (Supplementary Fig. 1d). During sorting, cells were negatively gated for CD31 and EpCAM/RET, before the remaining NP/iNP and SP/iSP population was separated on the basis of ITGA8 and PDGFRA (Fig. 2c, Supplementary Fig. 1e). Such sorted cells from E11.5 embryo (Supplementary Fig. 2a), E12.5 embryo (Supplementary Fig. 2b) and from differentiated EBs (Fig. 3) were devoid of any detectable endothelial or UB/ eUB contaminations. Sorted iSP cells were positive for FOXD1 and MEIS1 (Fig. 2d) [*n* = 3/3, 100% positive; CI: 83% to 100%]. The percentage of iNP and iSP obtained varied between differentiation batches (iNP: 15–40%, iSP: 40–70%, *n* = 24).

Differences between mESC-derived iNPs and iSPs and their ex-fetu counterparts were analysed by RNA sequencing. Supplementary Fig. L2 shows the 50 most differentially expressed genes in an iSP/ SP comparison. As for UB, the difference between ES-derived iSP cells and ex-fetu SP cells dominates over variation between samples of the same cell type. A Geneontology/ Panther analysis of biological processes associated with these most differential genes highlights the processes of hexose synthesis, muscle differentiation, and stress. It is interesting to note that hexose-induced stress accelerates muscle differentiation^39^, so these data may imply a different rate of progress or place on the pathway towards myogensis, which is one natural fate of stroma. The ex-fetu cells expressed higher levels of genes annotated to muscle differentiation (specifically *Plagl1*, *Maff*, *Atf*3 and *Klf5*) than the iSP cells. Supplementary Fig. L3 shows the 50 most differentially expressed genes in an iNP/ NP comparison: here there were no statistically significant results in a Geneontology/ Panther search for biological processes associated with differences.

### UB tips cooperate with sorted NP and SP cells to form embryonic kidney like higher order ex fetu organoids

**Figure 4:**
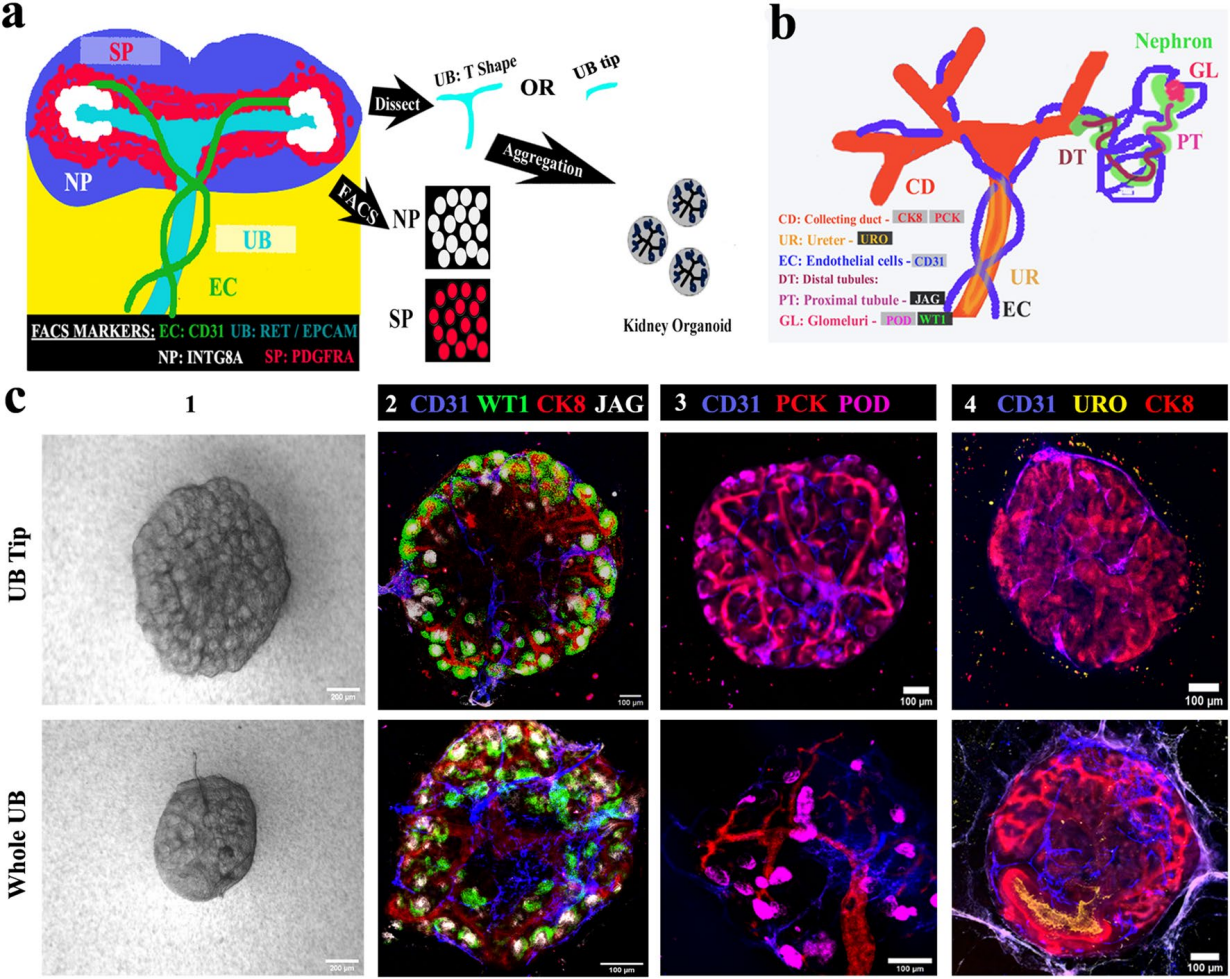
Ureteric bud tips contribute to ex-fetu organoids. (**a**) Schematic of the experiment: sorted nephron progenitor (NP) and stromal progenitors (SP) were aggregated with either whole UB (T shape) or UB tip. (**b**) Schematic summary of the natural expression of markers used in C. (**c**) (1) Bright field and (2–4) immunofluorescence images showing arborization of UB and formation of nephrons and epithelia, whether the organoids began with an intact UB or a UB tip. Scale bar, 200 µm for bright field images and others 100 µm. CD31: Platelet endothelial cell adhesion molecule, CK8: cytokeratin 8, WTI: Wilms tumor 1, JAG: jagged1, PCK: pan-cytokeratin, POD: podocalyxin, URO: uroplakin. Images are representative of at least three different experiments.

Our eUBs have entirely tip character, which makes them different from natural UBs. It is not known whether initial tip-stalk asymmetry of an UB is required for the organotypic anatomy of kidney organoids. To test this, we conducted the experiment depicted in Fig. 4a, with criteria for success explained in Fig. 4b. We initially tested ex-fetu NP and SP cell numbers/ratios ranging from 25,000 to 50,000 from both E12.5 and E13.5 embryos for their ability to form organoids. Since 35,000 of both cell types (from E12.5 embryos) worked well for us, this combination was used for all further experiments of this type. Aggregates of ex-fetu NP and SP cells with no UB failed to form anything recognizably kidney-like and died, as expected^40^ (Supplementary Fig. 3a) [*n* = 0/4, 0% made kidney-like organoids; CI: 0% to 13%]. Both ‘tip’ and ‘whole UB’ were able to form ex-fetu organoids with the mixed ex-fetu NP and SP cells, and the morphology of organoids formed was similar in bright-field microscopy, irrespective of whether the tip or the whole ex-fetu UB was used for aggregation (Fig. 4c 1). Both types of organoid had a single, branched ureteric bud tree (pan-cytokeratin, PCK and cytokeratin, CK8); proximal tubules (jagged 1); glomeruli (WT1); podocytes (podocalyxin); and endothelial cells (CD31) (Fig. 4c 2) [*n* = 4/4, 100% organoids showed these features; CI: 88% to 100%]. Nephrogenesis and endothelial cell formation was not affected by the choice of UB part. However, when whole ex-fetu UB was used, it formed a less dense tree than when tip was used (Fig. 4c 3). The ureteric bud tree formed had a more kidney-like shape (Fig. 4c 2,3) with the trunk expressing uroplakin (Fig. 4c 4) [*n* = 3/3, 100% positive; CI: 83% to 100%], showing its differentiation towards ureter.

### Separate testing of eUB and iSP cells in combination with embryo-derived components

**Figure 5:**
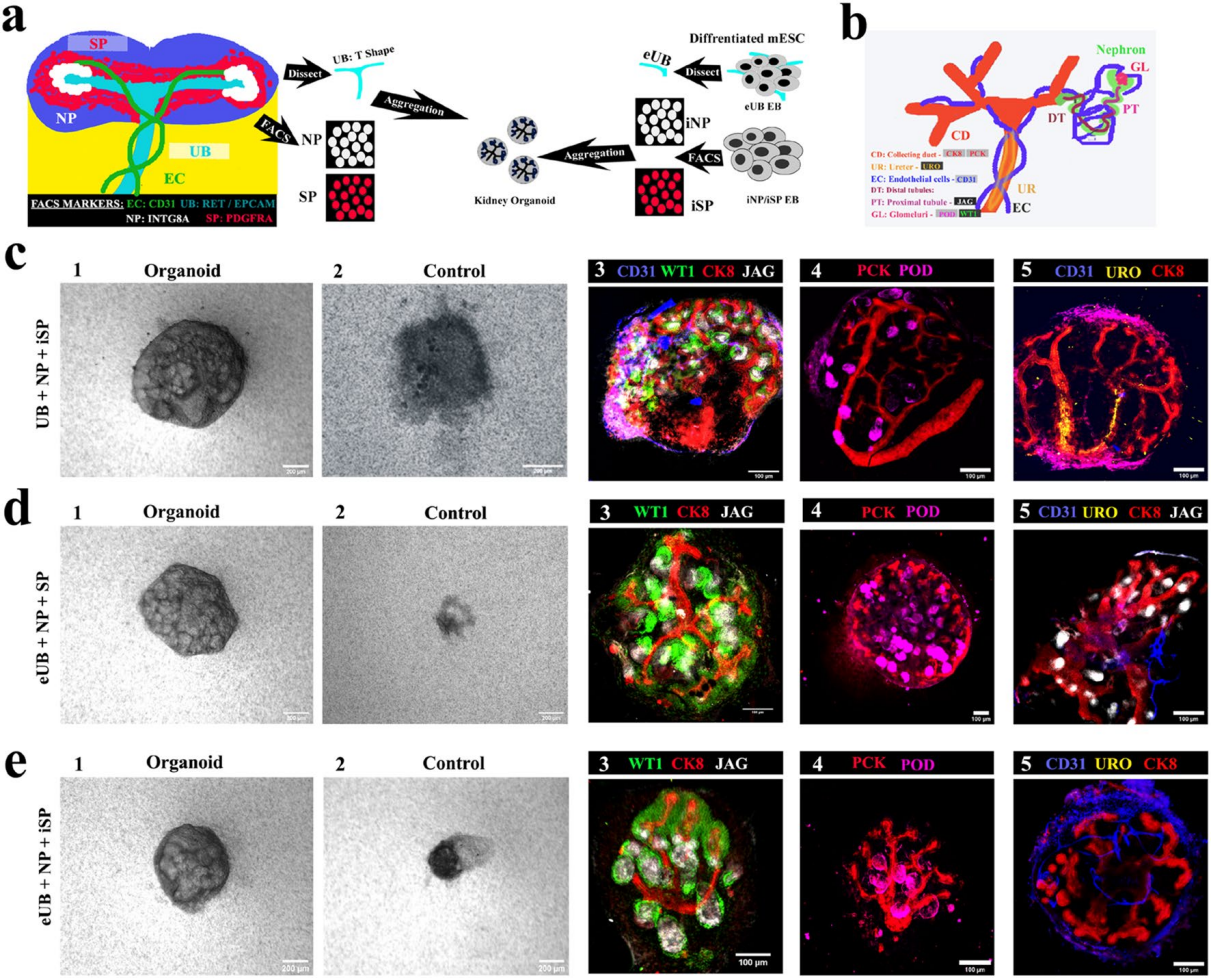
Testing mESC-derived components in chimaeric organoids. (**a**) Schematic of the experiment. (**b**) Schematic summary of natural expression of markers used in C-E. (**c**) Chimaeric organoids formed by aggregating whole UB (T shape) with sorted nephron progenitor (NP) and induced stromal cells (iSP) [UB + NP + iSP]. (**d**) Chimeric organoids formed by aggregating eUB with sorted nephron progenitor (NP) and induced stromal cells (SP) [eUB + NP + SP]. (**e**) Chimeric organoids formed by aggregating eUB with sorted nephron progenitor (NP) and induced stromal cells (iSP) [eUB + NP + iSP]. Representative bright field (first two images) and immunostaining images are shown. In all cases the ureteric bud (UB/eUB) arborized and all other components developed, but only the UB formed a ureter-type exit tube extending beyond the kidney (c4). Controls are aggregates without either eUB or UB. Scale bar, 200 µm for bright field images and 100 µm for fluorescence images. Abbreviations: CD31: platelet endothelial cell adhesion molecule, CK8: cytokeratin 8, WTI: Wilms tumor 1, JAG: jagged1, PCK: pan-cytokeratin, POD: podocalyxin, URO: uroplakin. Images are representative of at least three different experiments.

As an important quality control step, we verified the behaviour of our mESC-derived eUB and iSP in combination with ex-fetu renal progenitor tissues obtained directly from mouse embryos. The experiment is represented schematically in Fig. 5a. Chimeric organoids were generated by aggregating a mouse ex-fetu UB (T shape) or an eUB with one of the following combinations: NP & iSP cells (UB + NP + iSP); eUB, NP & SP (eUB + NP + SP) and eUB, NP & iSP (eUB + NP + iSP). The chimeric organoids were evaluated by cell-type-specific marker expression. A diagram of the expression of these markers in a normal embryonic kidney is provided in Fig. 5b.

In all the combinations that included UB or eUB, cellular aggregates developed to form an organoid (Fig. 5 c1,d1,e1) [n = 15/18, 83% developed; CI: 63% to 100%] while controls without UB/eUB failed to develop and eventually died (Fig. 5c2,d2,e2) [*n* = 0/3, 0% developed; CI: 0% to 17%]. Organoids without iSP showed very much reduced eUB branching (Supplementary Fig. 3c) [*n* = 0/3, 0% branched normally; CI: 0% to 17%], and so were not further evaluated.

In all chimeric organoids, a single, branched, pan-cytokeratin + , cytokeratin 8 + ureteric bud tree was observed (Fig. 5c–e). On day 2, SIX2 + NPs had coalesced around branch tips (Supplementary Fig. 3b 1) [*n* = 3/3, 100% showed this; CI: 83% to 100%]. By day 3–4 they underwent nephrogenesis to form WT1 + and jagged1 + tubules (S-shaped bodies) (Fig. 5c3–e3, Supplementary Fig. 3b 2) [*n* = 3/3, 100% showed nephrogenesis; CI: 83% to 100%]. Organoids also showed CD31 + endothelial cells and podocalyxin + podocytes by day 5 (Fig. 5 c4-e5, Supplementary Fig. 3b 3) [*n* = 3/3, 100% positive; CI: 83% to 100%].

Chimeric organoids generated using mouse ex-fetu UB showed UB branching in the kidney (Fig. 4c) with the trunk extending out from the kidney as a ureter, expressing uroplakin (Fig. 5 c5, See Supplementary Video 1 online) [*n* = 4/4, 100% uroplakin + ; CI: 88% to 100%], showing trunk differentiation towards ureter. However, organoids generated using eUB (eUB + NP + SP and eUB + NP + iSP) had crowded branches and showed no detectable uroplakin expression (Fig. 5 d5 & e5, See Supplementary Videos 2 & 3 online) [*n* = 0/4, 0% uroplakin + ; CI: 0% to 13%].

### Higher-order kidney organoids generated solely using mESC derived cells

The above sections were effectively quality-control steps toward our overall aim, to produce kidney organoids arranged around a single ureteric bud tree, entirely from mESC.

**Figure 6:**
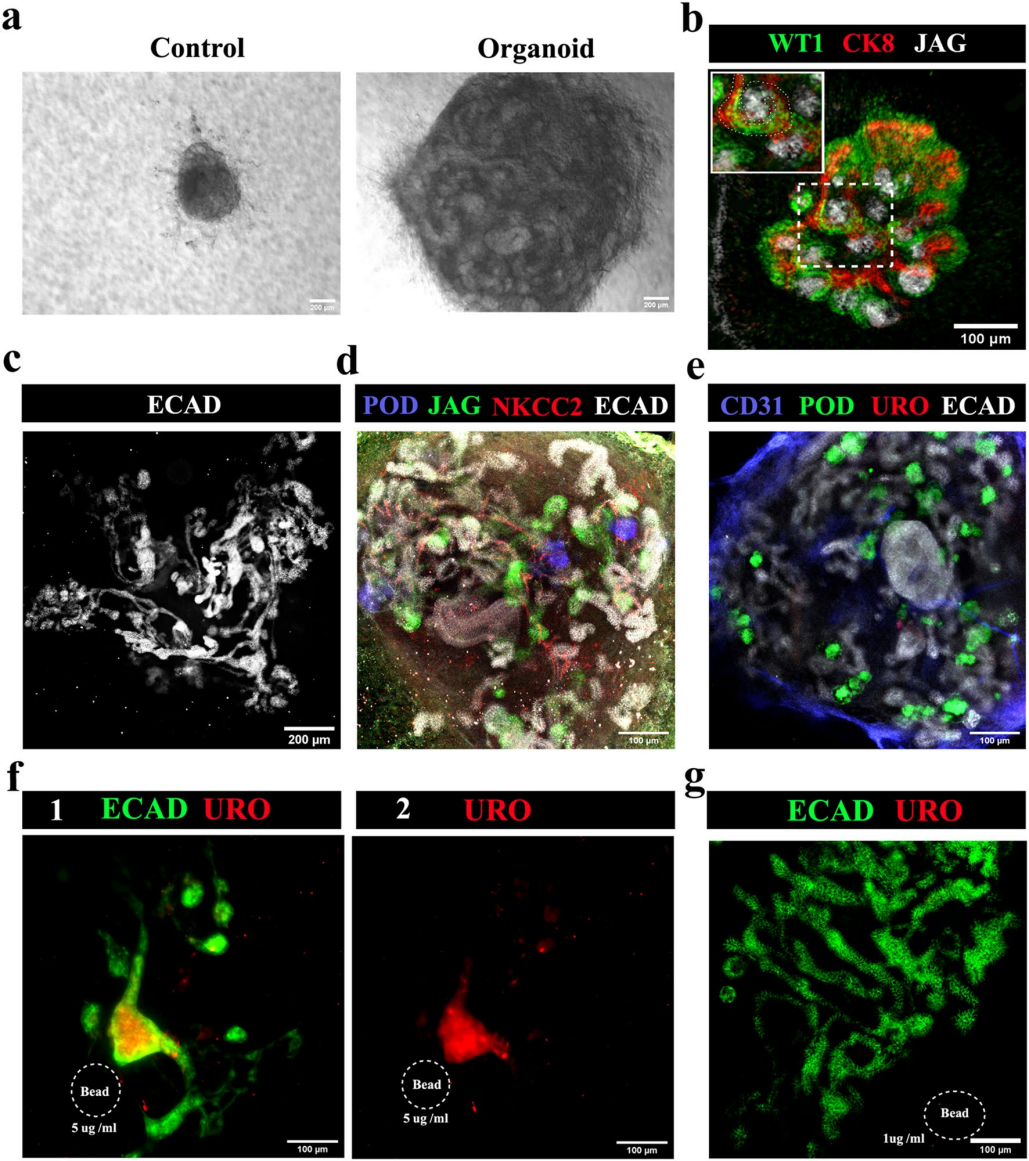
All-mESC organoids form nephrons connected to a ureteric bud/ collecting duct tree. (**a**) Bright field images of all-mESC-derived organoids, the control being an aggregate of iNP and iSP cells with no eUB: the iNP + iSP + eUB organoids grew (*n* = 15/18) while the controls did not (*n* = 0/3). Scale bar, 200 µm. (**b**) Early nephrogenesis around eUB branch tips at day 3 [an S shaped body is marked by a dotted line in the inset image] (*n* = 4/4), which grew and formed (**c**) a single connected ureteric bud-nephron tree structure by day 7 (*n* = 4/4). Scale bar: (**b**) 100 µm, (**c**) 200 µm. (**d**) Full-length nephron formation with podocytes, proximal tubules and distal tubules (n = 4/4); and (**e**) no uroplakin-positive ureter formation (*n* = 0/6) at day 10. Scale bar, 100 µm. (**f**) Uroplakin-positive ureter-like structure formation in the organoids induced by BMP4-soaked beads (5 µg /ml). Collecting duct-nephron tree is stained for ECAD. (1) Merged and (2) uroplakin channel images are shown. *n* = 2/6. Scale bar, 100 µm. (**g**) The lower concentration of BMP4 (1 µg/ml) did not induce uroplakin-positive structures (*n* = 4/4). Abbreviations: POD: podocalyxin (podocyte marker), JAG: jagged1 (proximal tubule marker), NKCC2: Na–K–Cl cotransporter 2 (distal tubular marker), ECAD: E cadherin (ureteric bud/distal tubular marker), CD31: platelet endothelial cell adhesion molecule (endothelial cells marker), URO: uroplakin (ureter marker), CK8: cytokeratin 8 (ureteric bud marker), WT1: Wilms Tumour 1 (podocyte/cap mesenchyme marker).

In all-mESC organoids, eUBs underwent branching morphogenesis within the aggregate [n = 15/18, 83% branched; CI: 63% to100%] while control cellular aggregates without an eUB failed to develop and eventually died (Fig. 6a) [*n* = 0/3, 0% survival; CI: 0% to 17%]. Nephrogenesis occurred and WT1 + and jagged1 + tubules (similar to S-shaped bodies) were observed by day 3 near the CK8 + ureteric bud tree tips (Fig. 6b) [*n* = 4/4, 100% showed nephrogenesis; CI: 88% to 100%], which later developed to form a single ureteric bud tree (Fig. 6c, See Supplementary Video 4 online) [*n* = 3/3, 100% made a single tree; CI: 83% to 100%]. By day 10, organoids showed the formation of more mature nephrons, with podocalyxin + podocytes, jagged1 + proximal tubules and NKCC2 + distal tubules (Fig. 6d, See Supplementary Video 5 online) [*n* = 4/4, 100% positive expression; CI: 88% to 100%]. The ureteric bud tree showed no expression of uroplakin (Fig. 6e) [*n* = 0/6, 0% showed uroplakin; CI: 0% to 8%].

To induce ureter formation, BMP4 soaked beads (5 µg/ml) were placed near the eUB branching structure, close to one branch. They inhibited branching near the bead and induced uroplakin expression. (Fig. 6f) [*n* = 2/6, 33% uroplakin expression; CI: 0% to 80%]. A lower concentration of BMP4 (1 µg/ml) did not affect the branching or induce uroplakin (Fig. 6g) [*n* = 4/4, 100% normal branching; CI: 88% to100%], while a higher concentration (15 µg/ml) completely destroyed organoid formation [*n* = 4/4, 100% inhibition of organoid formation; CI: 88% to100%] (data not shown).

**Figure 7:**
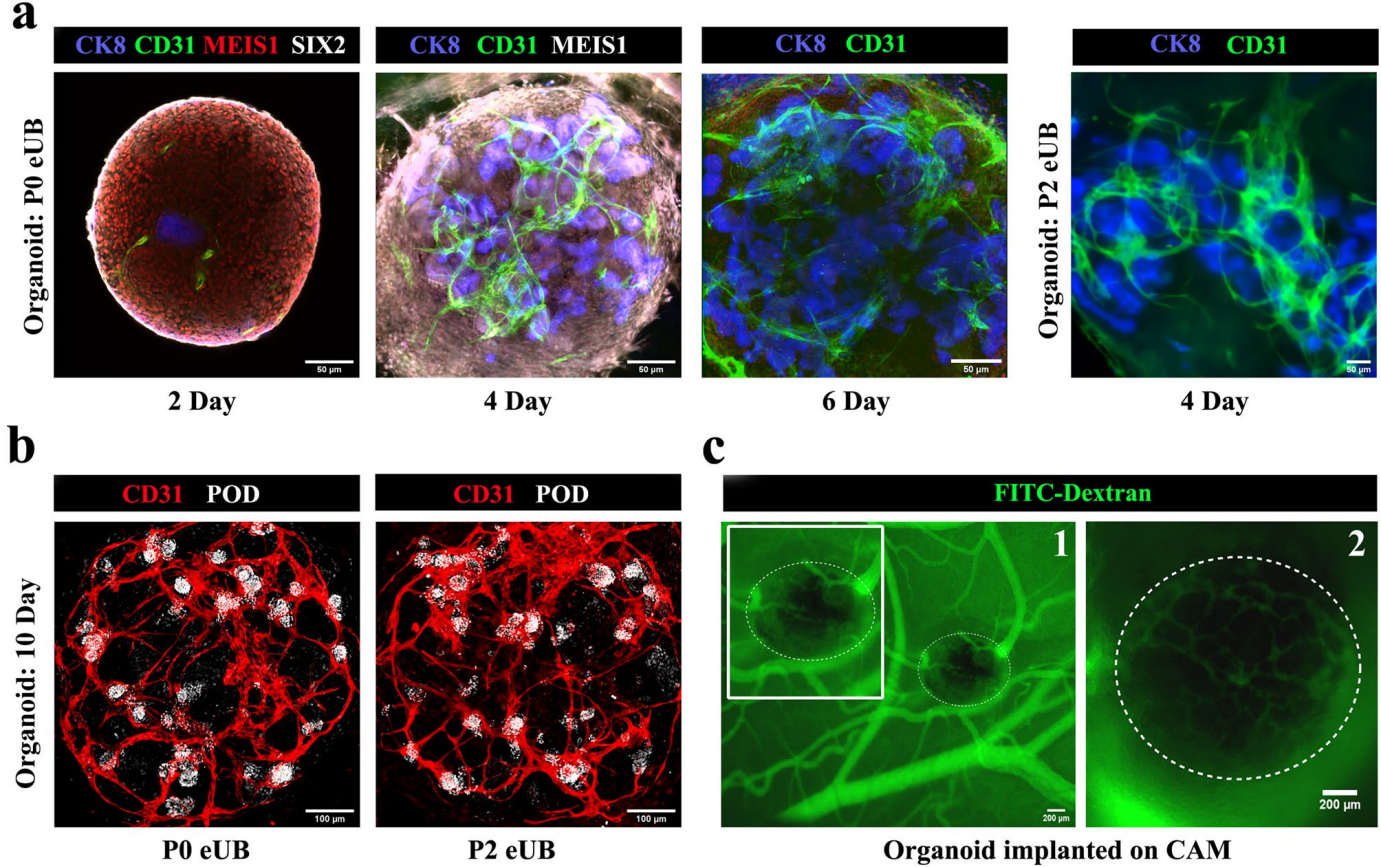
Small blood vessel formation in all-mESC organoid. (**a**) Blood vessel formation and its growth around ureteric bud branches in all-mESC organoids. *n* = 3/3. Scale bar, 50 µm (**b**) Immunostaining shows that vessels extend to the podocytes (white) at the proximal end of nephrons by day 10. Organoids generated using eUBs from differentiated embryoid bodies (Organoid: P0 eUB) and passage 2 eUB (Organoid: P2 eUB) were used for experiments. n = 4/4. Scale bar, 100 µm. (**c**) Fluorescence image of CAM implanted organoid after FITC dextran injection. (1) low and (2) high magnification images are shown. The organoid is marked with doted lines. Inset image shows the zoomed view of the organoid. *n* = 3/5. Scale bar, 200 µm. POD: podocalyxin (podocyte marker), JAG: jagged1 (proximal tubule marker), NKCC2: Na–K-Cl cotransporter 2 (distal tubular marker), ECAD: E cadherin (collecting duct / distal tubular marker), CD31: platelet endothelial cell adhesion molecule (endothelial cells marker), CK8: cytokeratin 8 (collecting duct marker), MEIS1: Meis homeobox 1 (stromal marker), SIX2: Six homeobox 2 (nephron progenitor marker).

All-mESC-derived organoids showed CD31 + endothelia close to the eUB by day 2. The endothelial population grew extensively and appeared to shadow ureteric bud branches (Fig. 7a), as in natural development^41^. By day 6 the endothelial cells formed a network (Fig. 7a) [*n* = 3/3, 100% showed a network; CI: 83% to 100%] and, by day 10, this extended to reach (within the resolution of the light microscopy used) the tight groups of podocytes (podocyte cluster) at the proximal end of nephrons (Fig. 7b, See Supplementary Video 6 online) [*n* = 4/4 organoids, 100% of organoids showed approach of endothelia to 'podocyte clusters'; CI: 88% to 100%]. Within each organoid 88% ± 7% of the total 'podocyte clusters' were approached closely by branches of the endothelial network] (Supplementary Video 7 online) [*n* = 6/6, 100%; CI: 92% to 100%]. Organoids made using passage 2 eUBs showed similar results (Fig. 7a&b) [*n* = 4/4 organoids, 100% showed approach of endothelia to 'podocyte clusters'; CI: 88% to 100%]. Within each organoid 81 ± 7% of the total 'podocyte clusters' were approached closely by branches of the endothelial network. The limitations of our light microscopic study mean that we can make no comment on the extent to which endothelia and podocytes might interact to make a glomerular filter.

To test whether organoids would attract host blood supply on transplantation, experiments were conducted using chicken egg chorioallantoic membranes (CAM). Day 4 all-mESC-derived organoids transplanted on CAM were invaded by chicken blood vessels, as shown by the presence of FITC-dextran injected through the chick vasculature within the transplanted organoids (Fig. 7c) [*n* = 3/5, 60% showed this; CI: 7% to 100%]. Organoids in culture maintained their endothelial networks up to day 10, after which they started to deteriorate.

## Discussion

Existing PSC differentiation methods can generate kidney organoids with many renal cell types, but they lack higher order structures, especially a ureter exit connected to a collecting duct tree^42^. Because of this drawback, organoids generated using existing methods are not suited for transplantation as an organ that produces urine and can drain it efficiently away. That is not to say that organoids have not proved useful. Mouse kidney organoids made from ex-fetu cells have been used to answer developmental questions such as whether nephrons are clonal or not (they are not^43^), the minimum number of cells required for nephrogenesis (about 5000^44^) and whether cells require specific genes such as *Lhx1* to incorporate into nephrons (they do^43^). Mouse kidney organoids made from PSC have been used to answer developmental questions such as whether notch signalling is required for proximal tubule formation (it is^45^), and to model drug-induced nephrotoxicity^45^. Human kidney organoids made from induced pluripotent stem (iPS) cells have been used to model human development^46^, and to model some human kidney pathologies from patient-derived iPS cells^47–49^ and from cells engineered to have genetic defects^48^. They have also been used to investigate critical pathways in renal repair^50^, and to model pathways of drug-induced nephrotoxicity^51^ and to screen for and report nephrotoxicity fluorescently^52^. It is important to note, though, that there are differences between human and mouse development^53^ and caution should be used in extrapolating from one to the other.

Despite the utility of existing organoids mentioned in the foregoing paragraph, anatomical organization of the kidney is critical for the organ to perform its normal physiological functions, primarily filtering blood to make urine and to pass this urine on to the bladder. Our lab has been working towards improving the organ-scale anatomy of organoids for a few years now and we have developed methods of producing kidney organoids that develop around a single ureteric bud/ collecting duct tree from ex-fetu cells^1,2^. Recently, Taguchi and colleagues were able to achieve this higher order organization (around a single collecting duct tree) in chimeric organoids^6^, and they and others^17^ have also made organoids from human iPS cells that represent only the ureteric bud/ collecting duct tree. However, production of a kidney with higher level organization using the Taguchi method relied on ex-fetu stroma and lacked endothelial blood vessels, even to the extent observed in other methods^54,55^. In this study, we have further improved the system, and were able to generate higher order kidney organoids solely using PSC-derived cells. Apart from the kidney-like anatomy with nephrons connected to a single ureteric bud tree, the organoids were complete with at least a small-vessel endothelial network.

We previously demonstrated that eUBs can form collecting duct or ureter depending on host mesenchyme^18,19^. In this study, we have further characterised these eUBs and shown that they can be propagated in vitro, without losing their functional properties. Such in vitro propagated eUB organoids can be very useful for modelling diseases such as polycystic kidney that affect the collecting duct branching morphogenesis.

For differentiating PSC to nephron progenitors, several protocols are available^4,5,7^. For this study, we adapted the method developed by Taguchi and colleagues^14^ to generate both cell types (iNP & iSP) in the same culture. It is likely that iNP & iSP cells generated using other differentiation protocols in a 2D monolayer culture^5,7^ would also work. They might even offer advantages, especially as 2D monolayer differentiations are more consistent between batches and are less work intensive compared to 3D embryoid body differentiation^56^. The iSP cells we generated (PDGFRA^High^/ITGA8^Low^) were functional, as demonstrated by their ability to support UB/eUB branching, nephrogenesis and formation of small vessels. As renal stroma consists of diverse populations^36,37^, it is not clear from our studies to which stromal population iSP cells belong, and further studies are needed to confirm their identity and how realistically they represent stromal cells in the actual embryo. However, our study is a first step, and is the first report of a protocol for generating functional renal stromal cells from PSC. Further optimizations may be needed for generating different types of stromal populations in a kidney.

Our adaptation of the differentiation protocol, with sorting, has allowed us to make, for the first time, renal organoids that show organization around a single ureteric bud tree, made entirely from PSC. This represents a significant 'catching up' of organoids made from PSC with those made from ex-fetu renogenic stem cells, and introduces an architecture appropriate for function. As a further step to catch up with the state-of-the art in organoids made from ex-fetu cells, we attempted to use the techniques of Mills and colleagues^2^, to induce ureter formation at one pole of the ureteric bud tree, using a local source of BMP4. BMP4 beads induced a uroplakin-positive ureter like-region at the bead end. It was, however, technically challenging to place BMP4 beads in the ‘all-mESC organoid’ due to their small size and 3D nature of the organoid. In most cases the growth of organoid was severely inhibited and eventually destroyed from the damage caused by implanting BMP4 bead. Nevertheless, it may be possible to improve success rate by using more advanced tools to deliver growth factors locally^57–59^. Uroplakin-positive ureter-like regions that were observed in whole UB and BMP4 treated all-mESC organoids lacked peristaltic contractions, probably due to the inability of the stromal cells (sorted ITGA8^Low^/PDGFRA ^High^) to differentiate into smooth muscle cells. A similar observation was reported in *Tbx18* knockout mice: these develop ureters with very thin surrounding mesenchyme, and fail to develop smooth muscles^60^. As we were only using metanephric mesenchymal stromal cells for the experiments, TBX18^+ve^ cells are less likely to be present in our organoids. Therefore we would be unlikely to have a reservoir of cells to generate smooth muscles needed for the peristaltic contractions. Development of these cells from pluripotent sources would be an important next step.

No published protocol is able to generate PSC derived kidney organoids with endothelial cells infiltrating the area of tubules to the extent seen here under in vitro static culture conditions^61^. Implanting organoids under kidney capsule^62,63^, on chick chorioallantoic membrane^4^, under skin^55^ or implanting them between arterial-venous flow^64^ has resulted in excellent vascularisation, but this is derived largely from the host. The all-mESC organoids we describe here develop a rich endothelial network in vitro, without the need for a host. The network formed in vitro did not, however, conform anatomically to that of a natural kidney and it is not clear how much of a problem this will be. We also do not know the extent to which these incoming vessels formed anatomical and physiological relationships with podocytes. Future approaches might culture the organoid in vivo, adjacent to a blood vessel capable of vascularizing a transplanted fetal kidney. This would allow these questions to be addressed in a more stable system (more stable in the sense of allowing longer term culture than CAM does, where the movements of the growing chick embryo create problems).

One drawback of the current technique is the complicated procedures involved in generating progenitor populations, which can limit the number of organoids that can be generated at a time. Our results show that eUBs can be propagated in vitro, and protocols for expanding iNPs are already available^65,66^. Developing a methods to expand stromal cells would help to simplify this organoid generation protocol. Immaturity is another drawback of the organoids, as evident by the lack of loop of Henle formation. It may be possible to induce loops of Henle by culturing them for a longer duration under low volume, again using techniques developed in the ex-fetu organoid system^67,68^.

We have improved the currently available kidney organoid generation methods. The all-mESC organoids, generated solely using PSC, had a kidney-like architecture, with a single ureteric bud tree having nephrons connected to it. They also showed formation of extensive endothelial network formation, with endothelial cells frequently approaching glomerular podocytes (whether this went on to result in formation of glomeruli and filtration membranes was not determined). This work is a step forward for developing mini kidneys suitable for functional transplantations, particularly in mice.

## Methods

Supplementary Table 1 and Supplementary Table 2 contain details of reagents and antibodies.

### Isolating mouse embryonic kidney stem cells

All procedures listed here were approved by the Institutional Animal Care and Use Committee of the University of Edinburgh, and were carried out in accordance with their guidelines and regulations. Pregnant CD1 mice were sacrificed between E11.5 and E13.5 by trained UK Home Office license holders in accordance with Schedule One of the UK Animals (Scientific Procedures) Act, 1986. Embryos were collected, decapitated, and their kidneys used freshly or stored (4 °C up to 3 days) in KCM (Advanced DMEM/F-12, 10% fetal bovine serum, 1 × GlutaMAX and 1 × antibiotic–antimycotic solution). For sorting, 25—60 kidneys were rinsed in PBS, the ureter being removed before making single cell suspension.

Intact UB tips or T-shaped whole UBs were isolated from E11.5 kidneys using tungsten needles (kidneys were treated with 0.25% trypsin–EDTA for 2 min before dissection). To maintain tip-stalk distinction, a thin layer of ureteric mesenchyme was retained in the distal part of whole UBs.

### Mouse embryonic stem cell culture and maintenance

mESC were cultured on 0.1% gelatin-coated 6-well plates in mESC culture medium (Glasgow Minimum Essential Medium with 10% FBS, 1 × GlutaMAX, 1 × MEM Non-Essential Amino acids, 1 × sodium pyruvate, 10 mM β-mercaptoethanol, with 1 U/μl leukaemia inhibitory factor). Cells were split using Accutase and plated at 1:5 or 1:10.

### Sox8-mCherry mESC reporter line generation

*pSox8-2A-mCherry* homology-directed repair template and *pSpCas9-2A-Gfp* vectors were used for generating the *Sox8-mCherry* mESC line (Supplementary Fig. 4). gRNA-encoding oligos CACCGACCACCCTGACCCGACCCTG and AAACCAGGGTCGGGTCAGGGTGGTC were cloned into *pSpCas9-2A-Gfp* as described elsewhere^69^. Each construct was transformed into NEB-5-α competent bacteria, ampicillin- resistant colonies were expanded, and plasmids were screened via restriction digestion and Sanger sequencing. IB10 mESC were co-transfected using Lipofectamine 3000. 48 h later, puromycin selection (5 μg/ml) was started and after 2 weeks resistant colonies were picked and PCR-screened. Stable transfection was confirmed by sequencing and mCherry expression.

### Induction of ureteric bud from embryonic stem cells

'Base medium’ was 75% Iscove’s modified Dulbecco’s medium: 25% Ham’s F-12, with 0.5 × N2, 0.5 × B27, 1 × antibiotic/antimycotic, 0.05% BSA, 0.5 mM L-ascorbic acid and 450 μM 1-thioglycerol. At 0 h, confluent mESC were dissociated with Accutase, seeded at 1,000 cells/well (U-bottomed, low cell-binding 96-well plates) and cultured in base medium (140 μl / well) to form EBs. Medium was replaced with fresh base medium containing specific growth factors as below. 48 h: 10 ng/ml human Activin A, 72 h: 0.3 ng/ml human BMP4 and 10 μM CHIR99021, 108 h: 0.2 μM retinoic acid, 100 ng/ml human FGF9, and 10 μM SB431542, 132 h: 0.2 μM retinoic acid, 100 ng/ml human FGF9, and 5 μM CHIR99021, 156 h: 10 µM Y27632, 0.2 µM retinoic acid, 1 µM CHIR99021, 5 ng/ml human FGF9, and 10% growth factor-reduced Matrigel, 180 h:'156 h' growth factors without FGF9, but with 3 µM CHIR99021 and 1 ng/ml GDNF, 204 h: ‘180 h medium’ with 2 ng/ml GDNF. Cells were cultured for a further 24 h, when budding, tubular structures (eUB) could be dissected manually for experiments.

In the original UB differentiation protocol^6^ EBs were dissociated at 48 h and at 150 h to sort cells. To make the protocol simpler, dissociation and sorting steps were skipped in our method.

### In vitro culture of ureteric bud

eUB were isolated from differentiated EBs (approximately 228 h) using hypodermic needles. P0 eUBs were cultured in branching medium (base medium with 0.1 μM retinoic acid, 100 ng/ml human Rspondin1, 1 ng/ml human GDNF, 100 ng/ml human FGF1 and 20% Matrigel). Buds from the branched structures formed were isolated every 4 -5 days and were cultured in fresh branching medium. For studying the influence of BMP4, eUBs were cultured in branching medium with 2.5 µg/ml mouse BMP4.

### Induction of nephron and stromal progenitors from embryonic stem cells

At 0 h, confluent mESC were dissociated with Accutase, seeded 1,000 cells/well (U-bottomed, low cell-binding 96-well plate) and cultured in base medium (140 μl / well) to form EBs. At different time points, medium was replaced with fresh base medium containing specific growth factors as below. 48 h: 5 ng/ml (E14 mESC) or 2.5 ng/ml (IB10 mESC) or 1 ng/ml (*Hoxb7-Gfp* mESC) of Activin A, 72 h: 10 μM CHIR99021, 108 h: 10 μM CHIR99021 and 10 µM Y27632, 132 h: 10 ng/ml human Activin A, 3 ng/ml human BMP4, 3 μM CHIR99021, 0.1 µM retinoic acid and 10 µM Y27632, 156 h: 1 μM CHIR99021, 5 ng/ml human FGF9 and 10 µM Y27632, 180 h: 1 μM CHIR99021 and 5 ng/ml human FGF9. Cells were cultured further 48 h and progenitor cells were sorted.

### Kidney organoid formation from sorted cells

Sorted progenitors were seeded at 35,000 cells/organoid in a U-bottom, 96 well plate and centrifuged (200 g, 3 min) to form cell sheets. Isolated eUB or UB was placed on top and cultured without any disturbance. After 48 h, organoids were transferred to 50% Matrigel- coated 12-mm, 0.4-μm-pore PET Transwell membranes (50 μl/ Transwell) at 37 °C for 3 min. 50 μl 50% Matrigel solution was then added on top, and organoids were incubated at 37 °C for 5 min. Warm KCM (370 μl/well) was added and the organoids were cultured for up to 12 days, with daily medium changes.

### Grafting of eUBs into cultured kidney rudiments

E11.5 kidney rudiments were cultured on 24-mm, 0.4-µm-pore Transwell membrane inserts, in KCM. *Hoxb7-Gfp* and *Sox8-mCherry* eUBs isolated from differentiated EBs (P0 eUB or P2 eUB) were grafted into metanephric mesenchyme or peri-Wolffian mesenchyme of E11.5 embryonic kidneys in culture as above. Kidney grafts were cultured for 3–4 days.

### Implantation of kidney organoids onto chick CAM

The experiment described here did not require any approval according to UK Animals (Scientific Procedures) Act, because of the young age of the chick embryos. Experiments were performed in accordance with relevant regulations and are reported in as per ARRIVE guidelines. Fertilized Hy-Line chicken eggs obtained from the National Avian Research Facility, University of Edinburgh, were incubated at 60% humidity and 38 °C (Brinsea Ova-Easy 190 Advance Series II I). After 24 h, 3 ml of albumin was removed using 18-gauge syringe. On day 6, a small window was opened on the egg surface using a scalpel blade and was sealed properly for preventing any contamination. Next day, through the window the surface of CAM was gently scraped and a 5-day old organoid was implanted. On day 10 a superficial CAM vein was injected with 1 mg/ml FITC-dextran and 5 min later implanted organoids were imaged using a fluorescence stereomicroscope (Leica, MSV269). For sacrificing, eggs were incubated on ice for 30 min and embryos were decapitated.

### Inducing ureter formation in organoids

For inducing ureter formation, BMP4-soaked beads were used as described elsewhere^2^. Briefly, Affi-gel blue beads washed twice with PBS were incubated in 40 μl mouse BMP4 (5 μg/ml) for 1 h at room temperature, rinsed and placed on organoids (day 3 'all-mESC organoids' in 6 well membrane insert) and were replaced daily. After 4 days, organoids were fixed and evaluated by immunostaining.

### Immunostaining

Samples were fixed in cold methanol for >  = 30 min or, for ITGA8, PDGFRA and RET, in 4% PFA. Samples were rinsed, blocked with 5% BSA, incubated overnight with primary antibody at 4 °C, rinsed and incubated overnight with secondary antibody at 4 °C. Samples were washed and dehydrated with 25%, 50%, 75% and 100% methanol. Ethyl cinnamate was added for clearing.

### Fluorescence-activated cell sorting

Volumes given below are for EBs from a 96 well plate or for a maximum of 75 kidneys. 5.5 µl of ITGA8-biotin antibody was added to 100 µl cell suspension and incubated for 10 min at room temperature. Cells were washed, centrifuged and resuspended in 100 µl medium. 1.5 µl of CD140a-APC (PDGFRA), 1.75 µl of RET, 1 µl of EpCAM, 1 µl of CD31 and 1 µl of PE-Streptavidin antibodies were added and incubated for 10 min at room temperature. Cells were washed, resuspended (750 µl 0.05% BSA) and stored on ice until sorting using a 4 laser FACSAria IIu SORP flow cytometer (BD, UK) with appropriate emission filters. ITGA8^High^/PDGFRA^Low^ NP/iNP, ITGA8^Low^/PDGFRA^High^ SP/iSP, RET^+ ^/ EpCAM^+^ UBs and EpCAM^+^/mCherry^+^ eUBs (from *Sox9-mCherry* line) were sorted.

### RNA sequencing

Kidneys from all the embryos of a mouse were pooled together as a sample, as were EBs from one batch of differentiation (E14 mESC for iNP / iSP differentiation; *Sox9-mCherry* for eUB differentiation). RNA from 35,000–100,000 sorted cells was isolated (RNeasy Plus micro kit) and quality was confirmed (Agilent bioanalyzer). 100 ng total RNA was used for library preparation (Illumina Stranded mRNA Prep kit protocol). Sequencing was performed using Novaseq and 50–64 million read-pairs were generated per sample. Reads were trimmed using Cutadapt (version cutadapt-1.18-venv) and aligned to the reference genome (GRCh38; from Ensembl) using STAR (version 2.7.3a). Raw count data were filtered and normalised. A principal component analysis and differential analysis was carried out using edgeR4 (version 3.28.1). The heat-maps shown in Supplementary Figs L1–L3 were produced using the ‘pheatmap’ package. In each figure, the range of the heatmap colours, from -1 to + 1, was anchored on the most divergent expression (maximum up, maximum down) in genes named in the heatmap figure. The scale is therefore local to each figure, and not the same between figures. Analysis of the differences that accompanies Supplementary Figure L1-L2 was done for the set of 50 genes appearing in the heatmap, using the Panther analytical tool at geneontology.org, with the *Mus musculus* genome as reference and a significance cut-off at false discovery rate (FDR) < 0.05. The same analysis was run for Fig L3, but no GO biological processes showed statistical significance.

### Statistical analysis

All images are representative, and the number of experiments conducted are mentioned in the figure legend. For categorical (feature present / absent) data, 95% confidence intervals (CI) were calculated using the binomial normal approximation interval corrected for small sample sizes as $p\pm 1.96\left[\sqrt{p\left(1-p\right)}/n\right]+1/2n$ ^70^. Lower and upper limit of the Confidence Interval was then expressed as percentage, considering the lowest possible as 0% and highest as 100%.

## Supplementary Information

Supplementary Information 1.

Supplementary Information 2.

Supplementary Information 3.

Supplementary Information 4.

Supplementary Information 5.

Supplementary Information 6.

Supplementary Information 7.

Supplementary Information 8.

Supplementary Information 9.

Supplementary Video 1.

Supplementary Video 2.

Supplementary Video 3.

Supplementary Video 4.

Supplementary Video 5.

Supplementary Video 6.

Supplementary Video 7.

## Data Availability

RNA-Seq datasets have been deposited on Gene Expression Omnibus (GEO) with accession number: GSE197139.
