# Supplementary figures and images for: Production of kidney organoids arranged around single ureteric bud trees, and containing endogenous blood vessels, solely from embryonic stem cells
Source: Sci Rep. 2022 Jul 22;12:12573. doi: 10.1038/s41598-022-16768-1 (PMC9307805; doi:10.1038/s41598-022-16768-1)

**a****E11.5  
Kidney**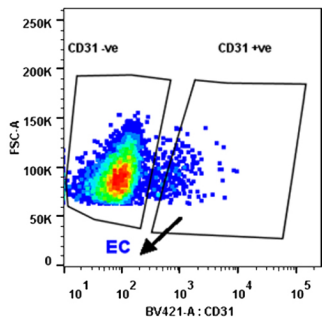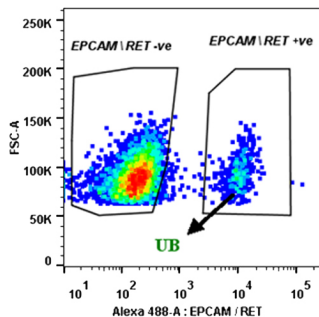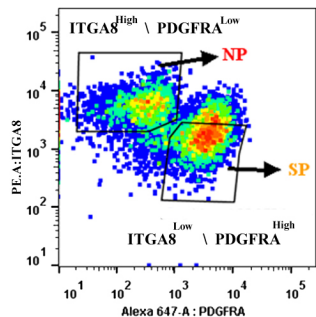**Sorted  
NP**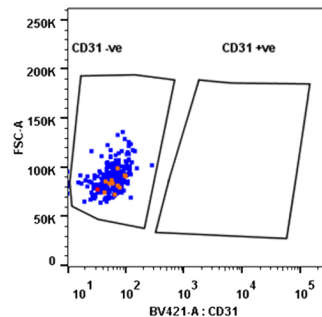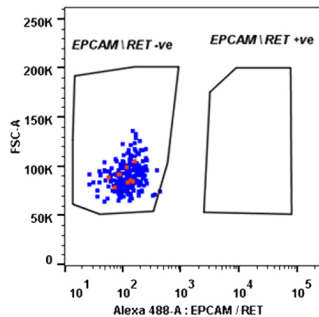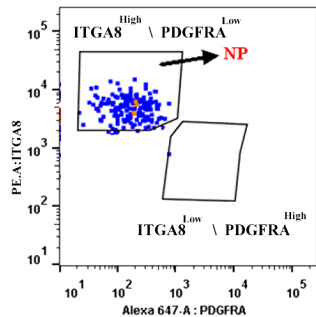**Sorted  
SP**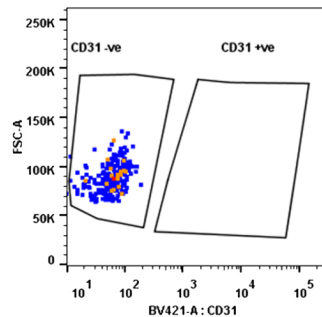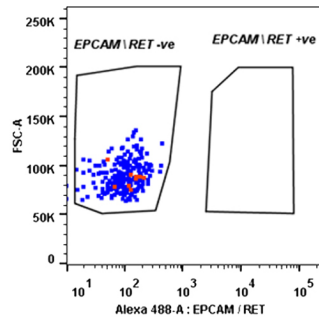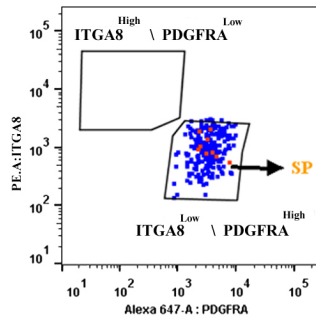**Sorted  
UB**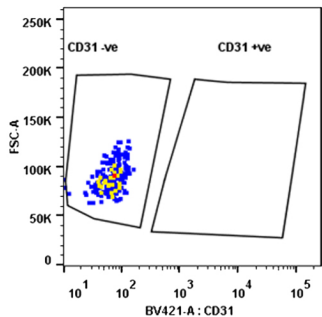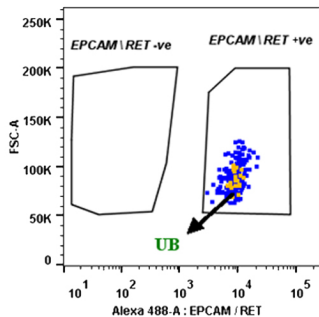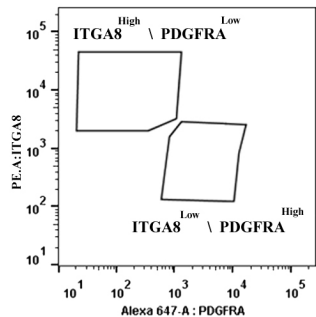

**b****E12.5  
Kidney**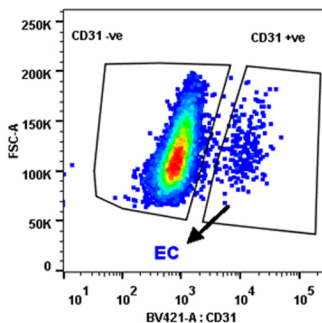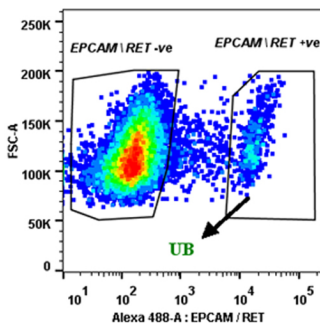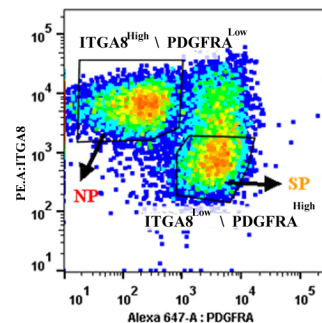**Sorted  
NP**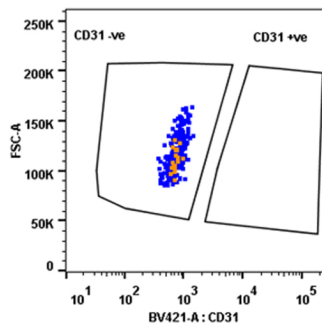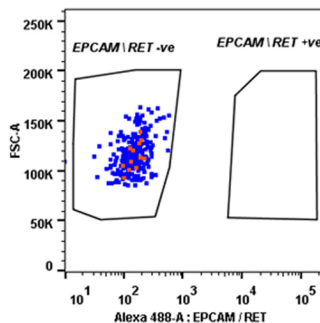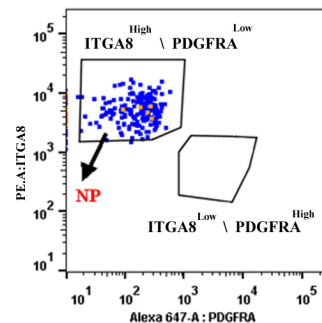**Sorted  
SP**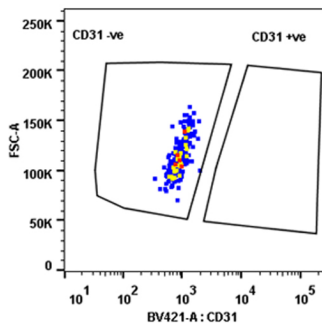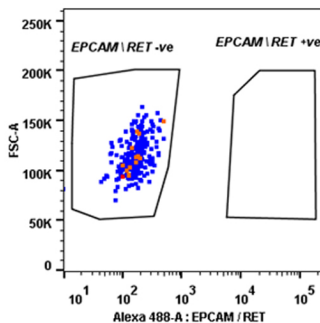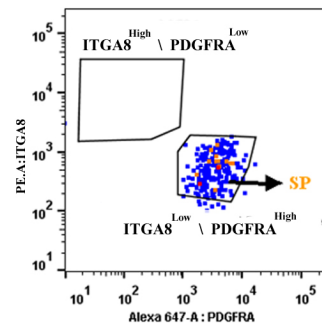**Sorted  
UB**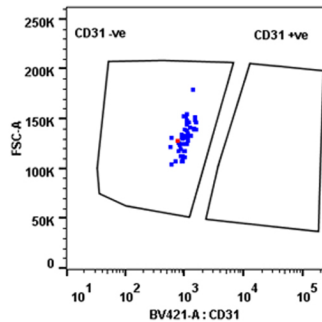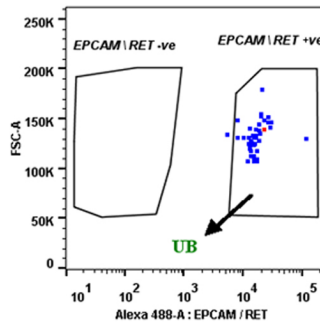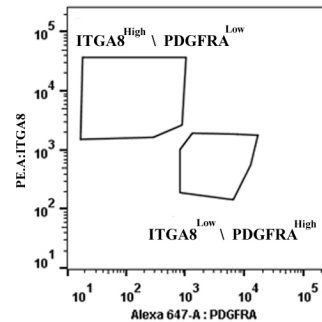

Supplement: Supplementary file 1 — Supplementary Information 1. [file 41598_2022_16768_MOESM1_ESM.pdf]

**a**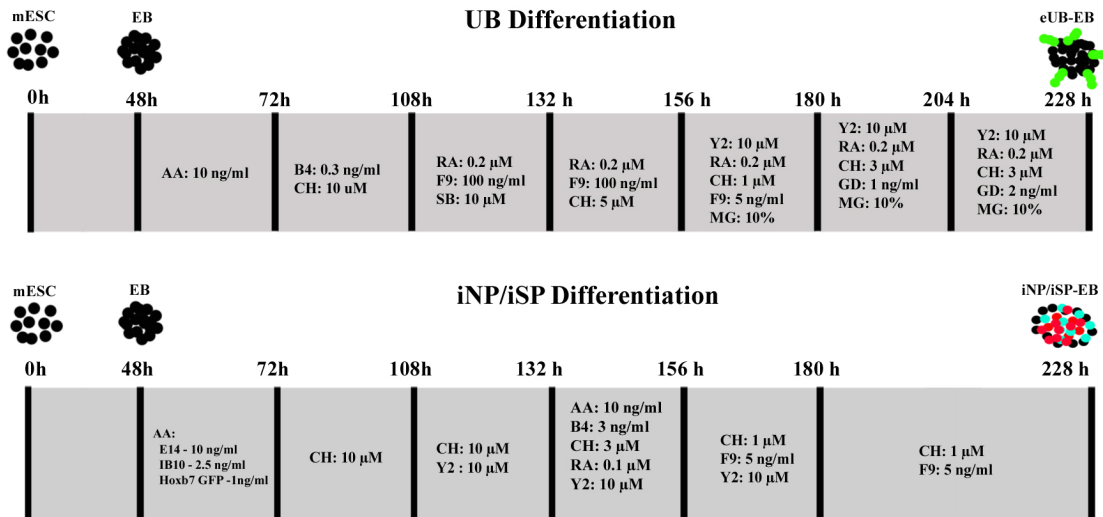**b****IB10*****Hoxb7-Gfp******Sox8-mCherry*****eUB-EB**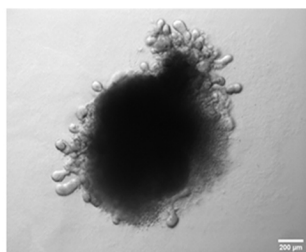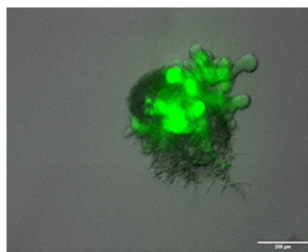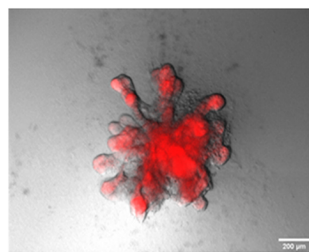**P4 eUB**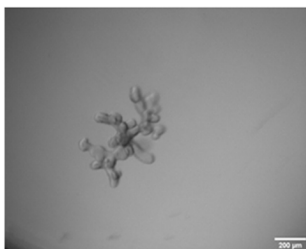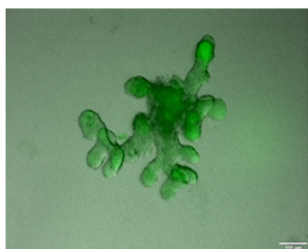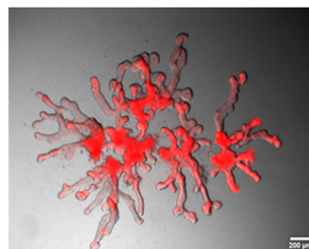

Supplement: Supplementary file 4 — Supplementary Information 4. [file 41598_2022_16768_MOESM4_ESM.pdf]

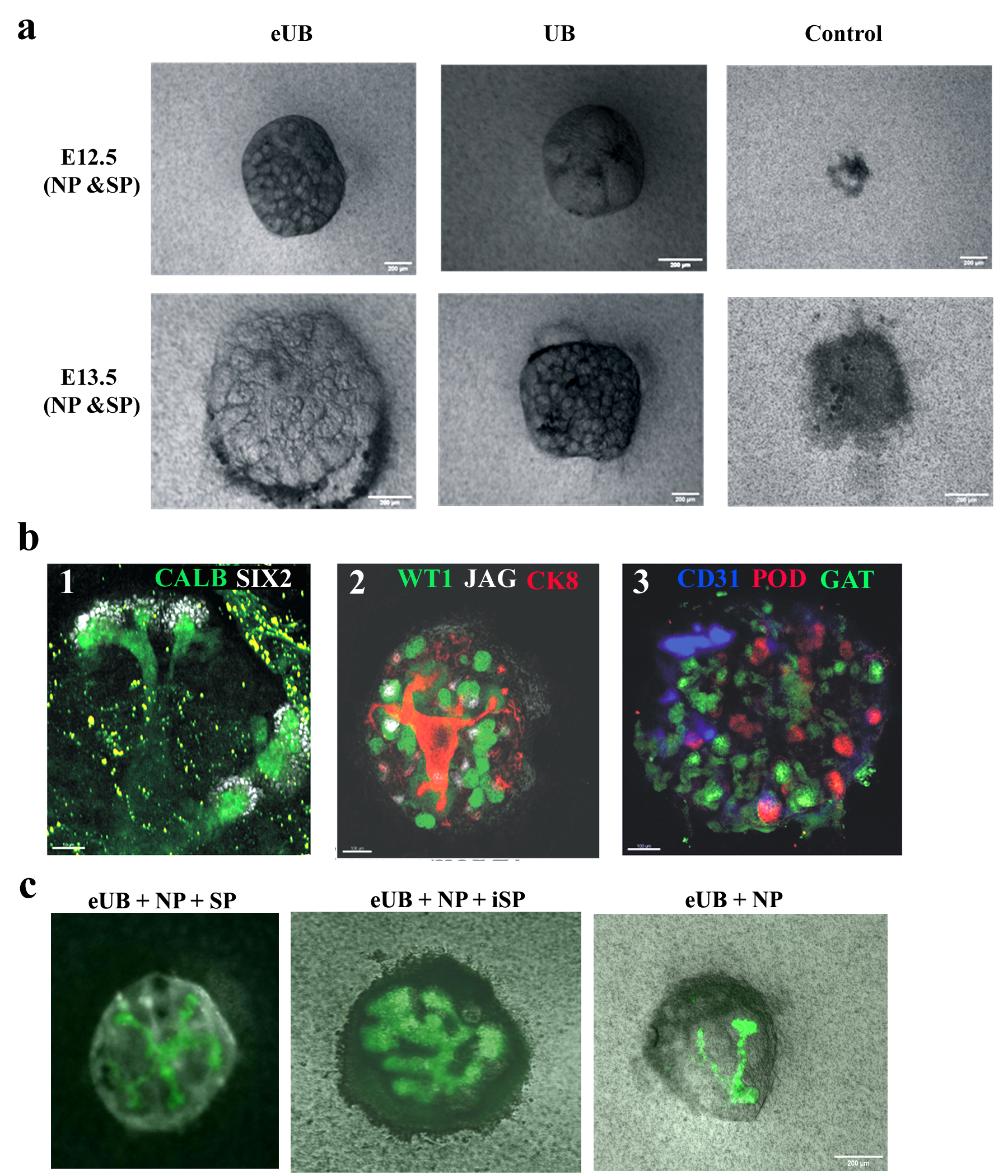

Supplement: Supplementary file 5 — Supplementary Information 5. [file 41598_2022_16768_MOESM5_ESM.tif]

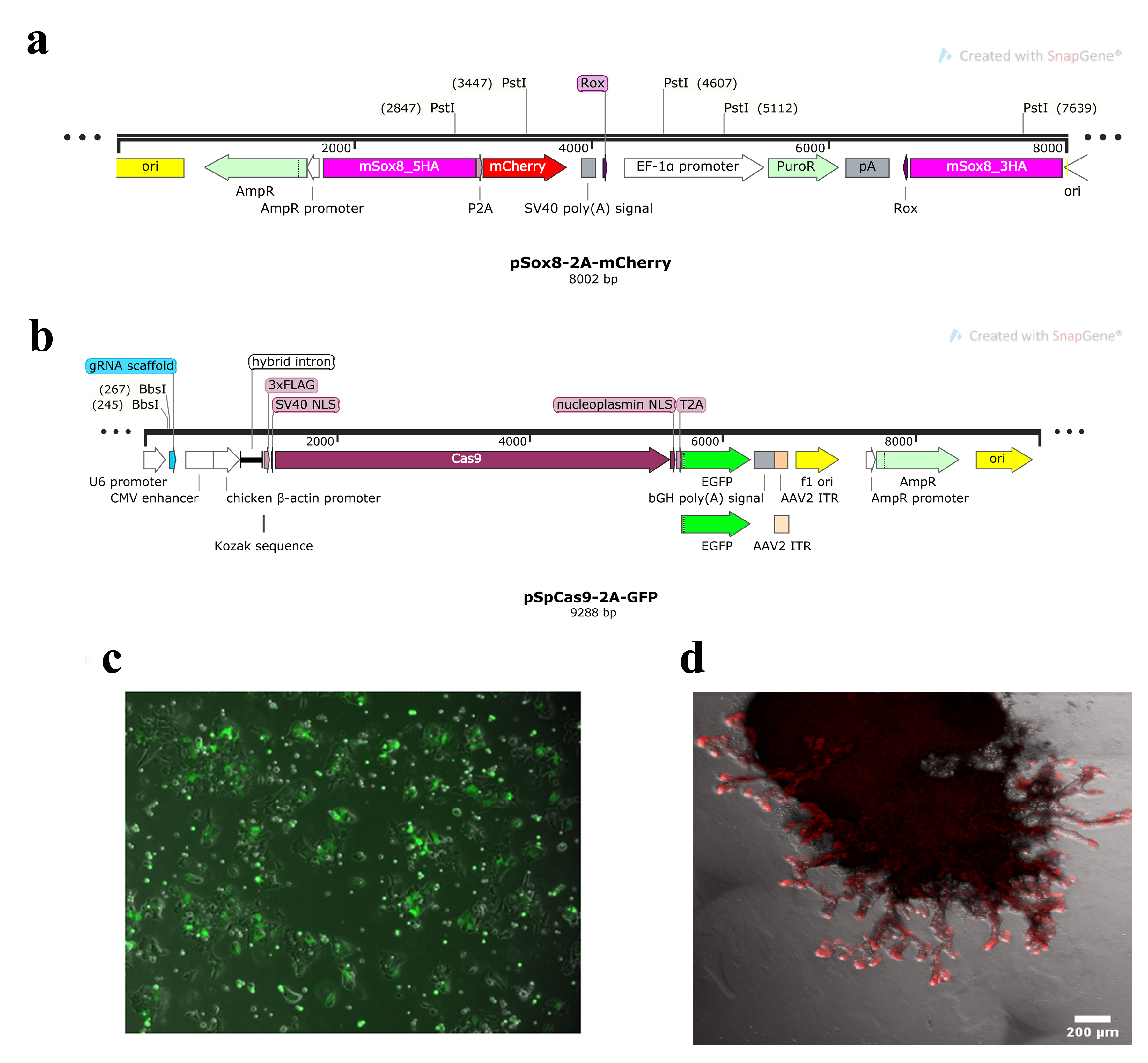

Supplement: Supplementary file 6 — Supplementary Information 6. [file 41598_2022_16768_MOESM6_ESM.tif]
