# Supplementary material for: Production of kidney organoids arranged around single ureteric bud trees, and containing endogenous blood vessels, solely from embryonic stem cells
Source: Sci Rep. 2022 Jul 22;12:12573. doi: 10.1038/s41598-022-16768-1 (PMC9307805; doi:10.1038/s41598-022-16768-1)
Supplement: Supplementary file 3 — Supplementary Information 3. [file 41598_2022_16768_MOESM3_ESM.docx]

**SUPPLEMENTAL INFORMATION LEGENDS**

**Supplementary Table 1: Details of media, reagents and growth factors**.

**Supplementary Table 2: Details of antibodies and lectin.**

**Supplementary Fig. 1: Differentiation, culture and isolation of progenitor cells. (a)**Time line diagram of the differentiation protocol. Abbreviations: AA: Activin A, B4: BMP4, CH: small molecule CHIR99021, RA: Retinoic acid, F9: FGF9, SB: small molecule SB431542, Y2: small molecule Y27632, MG: Matrigel and GD: GDNF. **(b) Propagation of eUBs in branching medium.** eUBs from parent differentiated embryonic bodies (eUB-EB) grew and branched when propagated in branching medium at least up to passage 4 (P4 eUB). eUBs from three different mESC lines; IB10, *Hoxb7-Gfp*, and *Sox8-mCherry*, were used for the experiments, as indicated in the row labels to the left of the micrographs. n=6/6 for each cell line. Scale bar, 200 µm. **(c) Standardisation of mESC differentiation to nephron-stromal progenitors.** Flow cytometry data showing the percentage of induced nephron progenitors (iNP) and stromal progenitors (iSP) obtained for different conditions during standardisation. Different concentrations of Activin A at 48 h; BMP4 and CHIR99021 at 72 h were tried for getting equal number of iNP and iSP populations from the same culture. Of the different combinations, the optimum found was selected (indicated by the arrow) for the experiments. A indicates Activin A, B indicates hBMP4 and C indicates CHIR99021. The number following the letter shows the concentration in ng/ml (for Activin A and hBMP4) or μM (for CHIR99021). For e.g. A2.5\B0.3C10 indicate treatment of 2.5 ng/ml Activin A at 48 h, followed by 0.3 ng/ml hBMP4 and 10 μM CHIR99021 at 72h. **(d) EpCAM expression in ureteric bud cells. (**E12.5 Kidney) Expression of EpCAM is restricted to ureteric bud cells of E12.5 embryonic kidneys. E-cadherin (ECAD) is expressed by both collecting duct and early nephrons. (eUB-EB) EpCAM expression by eUB in UB-pathway differentiated embryoid bodies. (iNP/iSP-EB) EpCAM expression is localized to ureteric bud-like cells (CK8+) and not expressed by nephron progenitors (SIX2) in nephron / stromal progenitor pathway-differentiated embryoid bodies. Scale bar, all images 200 µm. **(e) Contaminating cells in nephron-stromal progenitor population before modifying gating strategy.** Flowcytometry data of E11.5 kidney, E12.5 kidney and differentiated embryoid bodies (NP/SP Diff_­_ mESC) shows the presence of ureteric bud cells (EpCAM/RET +ve) and endothelial cells (CD31) in NP / iNP (ITG8A^high^ / PDGFRA^low^) and SP / iSP (ITG8A^low^ / PDGFRA^high^) gated cellular population. Ureteric bud (EpCAM/RET +ve) and endothelial cells (CD31) are marked as green and blue.

**Supplementary Fig. 2: Purity of sorted progenitors.** Purity of sorted progenitors from (a) E11.5 and (b) E12.5 kidney**.** The top row shows profiles of unsorted embryonic kidney cells with respect to the endothelial marker CD31, the ureteric bud markers EpCAM/RET, and the sorting profile based on ITG8A and PDGFRA. The second and third rows show post-sorting populations of NP and SP respectively: it can be seen that they are free from endothelial (EC: endothelial cells) and ureteric bud (UB) contamination. The last row shows the staining profile of sorted UBs with respect to RET/EpCAM, and the purity of the resultant sorted cells.

**Supplementary Fig. 3: Kidney organoid formation: (a) Bright field images of kidney organoids.** Organoids were reconstructed by aggregating 35,000 ex-fetu mixed nephron progenitors (NP) and stromal progenitors (SP) from E12.5 or E13.5 mouse embryonic kidney with ureteric bud (UB) or engineered ureteric bud (eUB). Organoids aggregated without UB or eUB were used as controls, and degenerated as expected. **(b) Anatomy of chimaeric organoids:** eUB + NP + SP chimeric organoids (1) on day 2 show the presence of SIX2-positive nephron progenitors around the tips of eUB branches (stained for calbindin D29k [CALB]). (2) On day 3, WT1-positive developing podocytes and jagged1 (JAG)-positive tubular cells can be seen, as can a ramifying CK8-positive collecting duct system (CK8). (3) On day 5, CD31-positive endothelial cells and podocalyxin-positive glomerular podocytes (POD) can be seen. The collecting duct system is stained for GATA3 (GAT). Scale bar: (1) 50 µm, others 100 µm. **(c) Functional ability of iSP to support eUB branching.** eUB (*Hoxb7-Gfp*) branching in different chimaeric organoids. Scale bar: 200 µm.

**Supplementary Fig. 4: *Sox8-mCherry* mESC line generation. (a)** Vector m­ap of *pSox8-2A-mCherry* homology-directed repair template. (**b**) Vector map of *pSpCas9-2A-Gfp* Sox8-cutting construct containing gRNA, Cas9 and GFP. **(c)** Live cell image of transfected IB10 mESC 48 h post-transfection. Many cells show GFP expression, indicating a successful transfection. **(d)** mCherry expression in ureteric buds from clone 29 (a selected clone after antibiotic selection, PCR genotyping and sequencing). _­_Clone 29 and Clone 43 were used for the experiments and are refereed as *Sox8*-*mCherry* mESC.

**Supplementary Fig. L1:** The 50 most differentially expressed genes, by FDR, between four samples of ex-fetu E11.5 ureteric bud and four eUB samples. The heat map, its -1 to +1 range normalized on the extremes of fold enrichment in this dataset, lists these genes, clearly showing most variation to be between the different sources of cells (ES-derived or ex-fetu) and not within each group. The table shows the biological process GO terms associated with these differences, only those with FDR < 0.05 being shown.

**Supplementary Fig. L2:** The 50 most differentially expressed genes, by FDR, between four samples of ex-fetu, sorted E11.5 stromal progenitor cells and four iSP samples. The heat map, its -1 to +1 range normalized on the extremes of fold enrichment in this dataset, lists these genes, clearly showing most variation to be between the different sources of cells (ES-derived or ex-fetu) and not within each group. The table shows the biological process GO terms associated with these differences, only those with FDR < 0.05 being shown.

**Supplementary Fig. L3:** The 50 most differentially expressed genes, by FDR, between four samples of ex-fetu, sorted E11.5 nephron progenitor cells and four iNP samples. The heat map, its -1 to +1 range normalized on the extremes of fold enrichment in this dataset, lists these genes, clearly showing most variation to be between the different sources of cells (ES-derived or ex-fetu) and not within each group.

**Supplementary Video 1: Z-stack of UB+NP+iSP chimeric organoid (day 7) showing ureter formation.** Ureteric bud tree (CK8) having a ureter like structure (URO) at one end can be observed. Endothelial cells (CD31) can also be seen. Abbreviations: CK8: cytokeratin 8, CD31: platelet endothelial cell adhesion molecule, URO: uroplakin.

**Supplementary Video 2: Z-stack of eUB+NP+SP chimeric organoid (day 7) showing no ureter formation.** Ureteric bud tree (CK8) without a ureter like structure (URO) can be observed. Early nephron tubules (JAG) can be seen. Abbreviations: CK8: cytokeratin 8, JAG: jagged1, URO: uroplakin.

**Supplementary Video 3: Z-stack of eUB+NP+iSP chimeric organoid (day 7) showing no ureter formation.** Ureteric bud tree (CK8) without a ureter like structure (URO) can be observed. Endothelial cells (CD31) can be seen. Abbreviations: CK8: cytokeratin 8, CD31: platelet endothelial cell adhesion molecule, URO: uroplakin.

**Supplementary Video 4: Z-stack of all-mESC organoid (day 10) showing connected ureteric bud–nephron tree.** E cadherin (ECAD) was used to stain both ureteric bud/collecting duct and distal tubules.

**Supplementary Video 5: Z-stack of all-mESC organoid (day 10) showing developed nephrons.** Nephrons with glomerular podocytes (POD), proximal tubules (JAG) and distal tubules (NKCC2 and ECAD) can be observed; and were connected to the ureteric bud tree (ECAD). Abbreviations: POD: podocalyxin, JAG: jagged1, NKCC2: Na-K-Cl cotransporter 2, ECAD: E cadherin.

**Supplementary Video 6: Z-stack of all-mESC organoid (day 10) showing small blood vessels.** Extensively grown endothelial cells (CD31) connecting glomerular podocytes (POD) can be observed. Abbreviations: CD31: platelet endothelial cell adhesion molecule, POD: podocalyxin.

**Supplementary Video 7: 3D video of a glomerulus in all-mESC organoid (day 10).** Endothelial vessel (CD31) infiltrated into glomerular podocyte clusters (POD) can be observed. Abbreviations: CD31: platelet endothelial cell adhesion molecule, POD: podocalyxin.
