# Supplementary material for: Production of kidney organoids arranged around single ureteric bud trees, and containing endogenous blood vessels, solely from embryonic stem cells
Source: Sci Rep. 2022 Jul 22;12:12573. doi: 10.1038/s41598-022-16768-1 (PMC9307805; doi:10.1038/s41598-022-16768-1)
Supplement: Supplementary file 7 — Supplementary Information 7. [file 41598_2022_16768_MOESM7_ESM.pdf]

Supplementary Fig L1

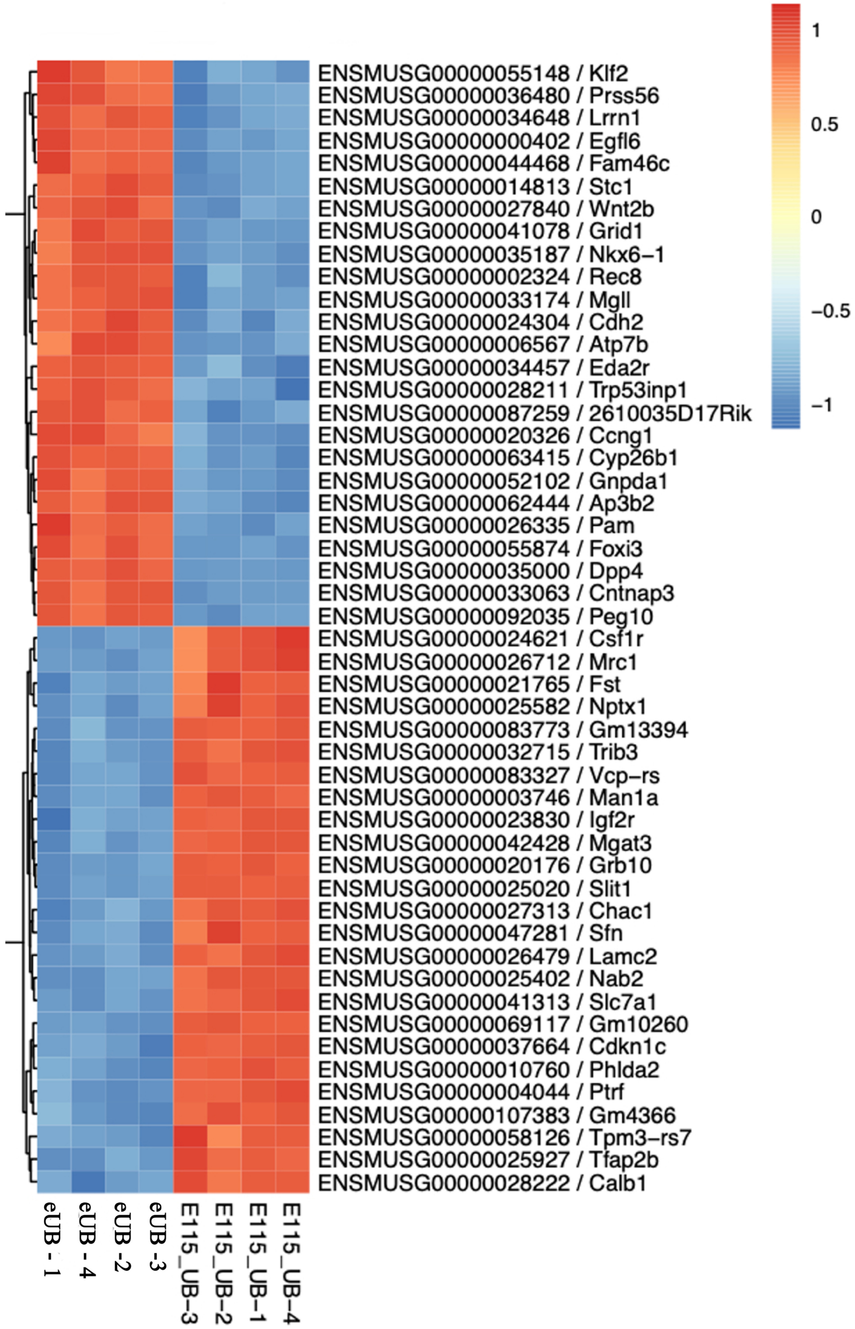

Biological Process	Fold Enrichment	+/-	FDR
1054 animal organ morphogenesis	5.09	+	0.0185
1096 regulation of locomotion	4.90	+	0.0243
1537 regulation of multi-cellular organismal development	4.19	+	0.0170
3597 cell differentiation	3.13	+	0.000916
3785 system development	2.98	+	0.00157
3352 regulation of cell communication	2.88	+	0.0140
3360 regulation of signalling	2.87	+	0.0134
6522 positive regulation of biological process	2.14	+	0.0123

Supplementary Fig L2

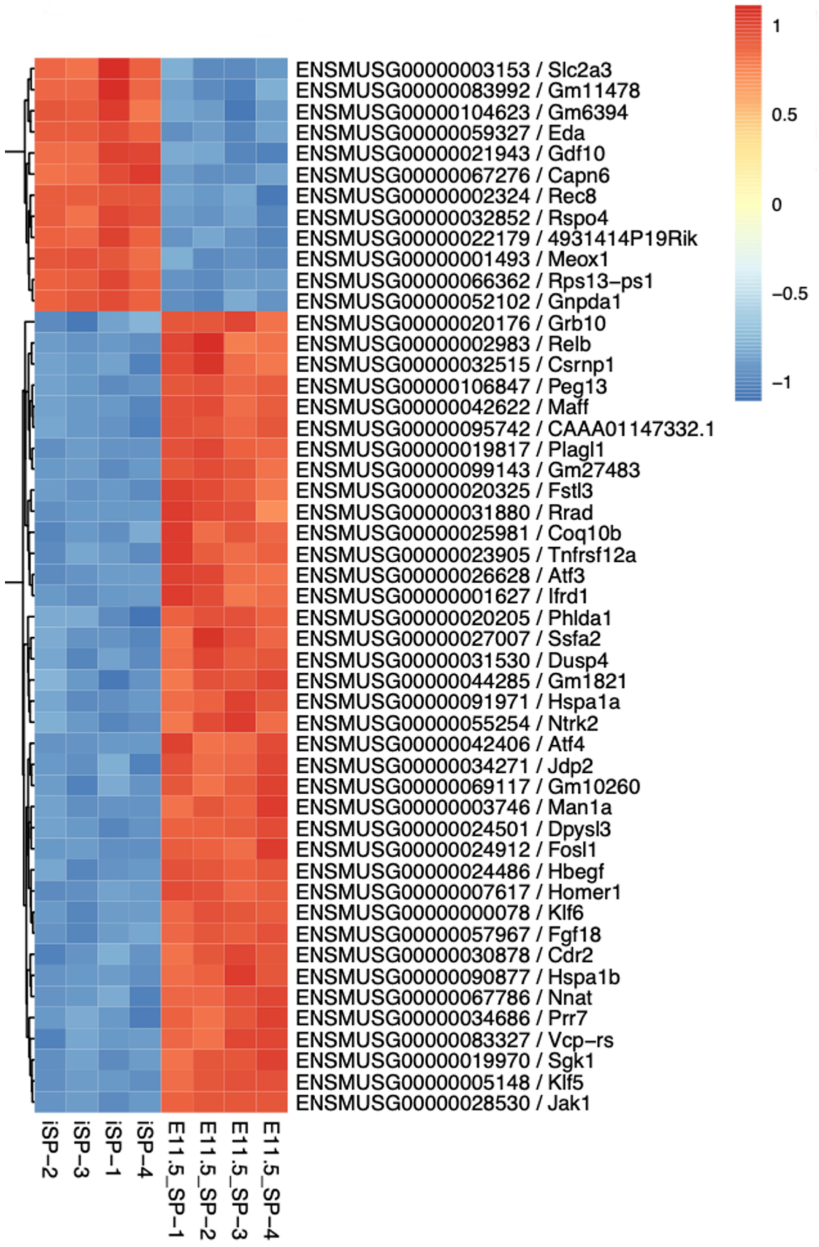

Biological Process	Fold Enrichment	+/-	FDR
36 hexose biosynthesis process	49.54	+	0.0232
60 skeletal muscle differentiation	39.63	+	0.00523
74 cellular response to unfolded protein	32.14	+	0.00865
369 transmembrane receptor protein kinase signalling pathway	9.67	+	0.0228
1615 positive regulation of transcription by RNA polymerase II	4.05	+	0.0264
1675 regulation of cell differentiation	4.26	+	0.0273
1787 positive regulation of cell communication	3.66	+	0.0476
1794positive regulation of signalling	3.65	+	0.0481
3785 system development	2.67	+	0.0256

Supplementary Fig L3

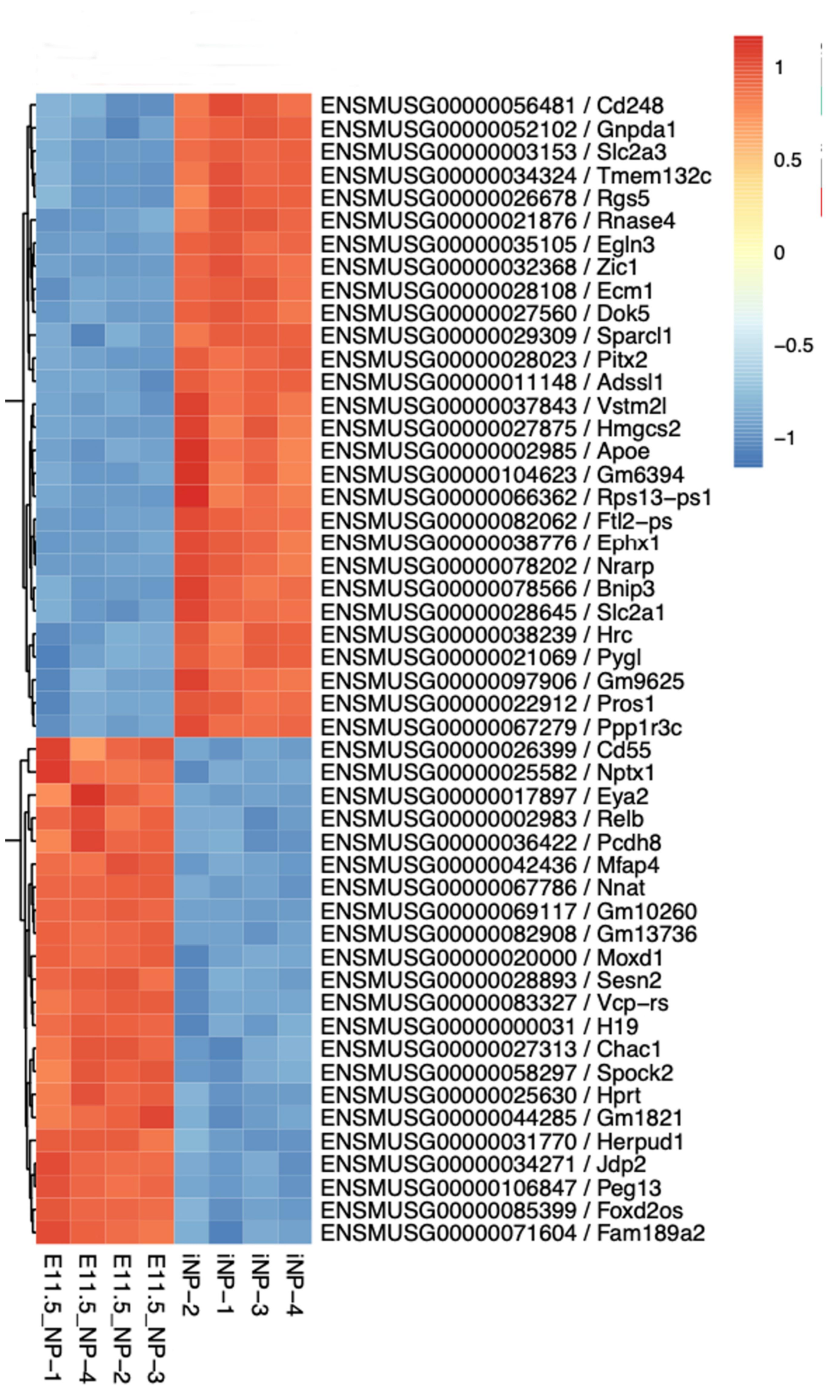

Analysis of these data for Biological Process GO terms reveal no associates significant at FDR < 0.05. That is why there is no table of GO terms associated with the heat map to the left.
