# Supplementary material for: Production of kidney organoids arranged around single ureteric bud trees, and containing endogenous blood vessels, solely from embryonic stem cells
Source: Sci Rep. 2022 Jul 22;12:12573. doi: 10.1038/s41598-022-16768-1 (PMC9307805; doi:10.1038/s41598-022-16768-1)
Supplement: Supplementary file 8 — Supplementary Information 8. [file 41598_2022_16768_MOESM8_ESM.docx]

| - **Reagent** | **Supplier** | **Catalogue number** |
| --- | --- | --- |
| **Peptides, Recombinant Proteins and Small Molecules** | | |
| Activin A | R&D | 338-AC-010 |
| CHIR99021 | Tocris | 4423 |
| hBMP4 | R&D | 314-BP-010 |
| hFGF1 | R&D | 232-FA-025 |
| hFGF9 | R&D | 273-F9-025 |
| hGDNF | R&D | 212-GD-010 |
| Human R Spondin 1 | R&D | 4645-RS |
| LIF | Santa Cruz | sc4989A |
| mBMP4 | R&D | 5020-BP-010 |
| Retinoic Acid | Sigma | R2625 |
| SB431542 | Tocris | 1614 |
| Y27632 (Rock Inhibitor) | Tocris | 1254 |
| **Media** | | |
| Advanced DMEM/F-12 | ThermoFisher | 12634010 |
| Glasgow Minimum Essential Medium | Sigma | G5154 |
| Ham’s F-12 | ThermoFisher | 1765054 |
| Iscove’s modified Dulbecco’s medium | ThermoFisher | 12440-046 |
| **Reagents** | | |
| 0.25% Trypsin-EDTA | ThermoFisher | 25200056 |
| 1-thioglycerol | Sigma | M6145-25ml |
| Accutase | ThermoFisher | A1110501 |
| Affi-Gel Blue beads | Bio-Rad | 1537302 |
| Antibiotic/Antimycotic | ThermoFisher | 15240-096 |
| B27 Supplement without Vita A | ThermoFisher | 12587-010 |
| b-Mercapto ethanol | ThermoFisher | 31350-010 |
| BSA powder | Sigma, | A9646 |
| BSA, 7.5% solution | ThermoFisher | 15260-037 |
| DPBS | ThermoFisher | 14190-094 |
| Ethyl cinnamate | Sigma | 112372 |
| FITC-dextran | Sigma | FD-2000S |
| Foetal bovine serum | HyClone | SV30160.03 |
| Gelatin | Sigma | G1890 |
| GlutaMAX | ThermoFisher | 35050-38 |
| L-Ascobic Acid | Sigma | A4403 |
| Lipofectamine 3000 | ThermoFisher | L3000001 |
| Matrigel | Corning | 354230 |
| MEM Non-Essential Amino acids | ThermoFisher | 11140-035 |
| N2 Supplement | ThermoFisher | 17502-048 |
| Puromycin | Sigma | 540411 |
| Sodium pyruvate | ThermoFisher | 11360-039 |

| **Plasticware** | | |
| --- | --- | --- |
| Low Binding, U bottom, 96 well plates | Greiner Bio | 650970 |
| Transwell membrane inserts - 12 well | Corning | 3460 |
| Transwell membrane inserts - 6 well | Corning | 3450 |
| 12 well plates | Corning | 3513 |
| 6 well plates | Corning | 3516 |
| Cell strainer (40 µm) | ThermoFisher | 22363547 |
| 12 well plates | Corning | 3513 |
| **Molecular Kits** | | |
| High Sensitivity DNA Kit | Agilent | 5067-4626 |
| HiFi DNA Assembly mastermix | NEB | M5520A |
| Illumina Stranded mRNA Prep kit | Illumina | 20040532 |
| Monarch gel extraction kit | NEB | T1020S |
| Monarch plasmid miniprep kit | NEB | T1010S |
| RNA 6000 Nano Kit | Agilent | 5067-1511 |
| RNeasy plus micro kit | Qiagen | 74034 |
| Q5 Hot Start High-fidelity master mix | NEB | M0494S |
| QIAshredder | Qiagen | 79656 |
| **Plasmids and Competent cells** | | |
| *pSpCas9-2A-GFP* | Addgene | 48138 |
| NEB-5-α chemically competent bacteria | NEB | C2987H |
| **Software and Algorithms** |  |  |
| FlowJo | Tree Star | https://www.flowjo.com |
| Image J | NIH | https://imagej.nih.gov |

**Supplementary Table 1: Details of mediums, reagents and growth factors**.
