# Supplementary material for: Production of kidney organoids arranged around single ureteric bud trees, and containing endogenous blood vessels, solely from embryonic stem cells
Source: Sci Rep. 2022 Jul 22;12:12573. doi: 10.1038/s41598-022-16768-1 (PMC9307805; doi:10.1038/s41598-022-16768-1)
Supplement: Supplementary file 9 — Supplementary Information 9. [file 41598_2022_16768_MOESM9_ESM.docx]

|  | **Supplier** | **Catalogue number** | **Working dilution** |
| --- | --- | --- | --- |
| **Primary antibodies** | | | |
| Calbindin D28k | Novus | NBP2 | 1:200 |
| CD140a (PDGFRA)-APC conjugated | BioLegend | 135908 | 1.5 :100 |
| CD31 | R&D | AF3628 | 1:200 |
| CD31-BV421 conjugated | BioLegend | 102423 | 1:100 |
| CD326 (Ep-CAM) 488 | BioLegend | 118210 | 1:100 |
| CK8 | DSBH repository | TROMA-1 | 1:200 |
| Dolicous biflourus agglutinin  (fluoresceinated) | Vector | FL-1031 | 1:500 |
| E-cadherin | BD | 610182 | 1:100 |
| FOXD1 | ThermoFisher | PAS-35145 | 1:50 |
| GATA3 | R&D | AF2605 | 1:200 |
| ITG8A-biotinylated | R&D | BAF4076 | 5.5 :100 |
| Jagged 1 | R&D | AF599 | 1:200 |
| MEIS11/2/3 | Active Motif | 39796 | 1:250 |
| NKCC2 | Proteintech | 18970-1-AP | 1:50 |
| Pan-Cytokeratin | Sigma | C2562 | 1:200 |
| Podocalyxin | R&D | MAB1556 | 1:200 |
| Ret | Santa Cruz | sc-167 | 1:25 |
| Ret-488 Conjugated | Santa Cruz | sc-101423 AF488 | 1.75 :100 |
| Six2 | Proteintech | 115621-1-AP | 1:50 |
| Sox9 | Sigma | AB5535 | 1:200 |
| Uroplakin | Gift from Tung-Tien Sun, New York University | | 1:500 |
| Wnt7b | Sigma | SAB2701193 | 1:1000 |
| WT1 | Abcam | ab89901 | 1:200 |
| **Secondary antibodies** | | | |
| Anti-Goat 488 | Invitrogen | A-11055 | 1:250 |
| Anti-Goat 594 | Invitrogen | A-11058 | 1:250 |
| Anti-Goat 647 | Invitrogen | A-21447 | 1:250 |
| Anti-Mouse 488 | Invitrogen | 21202 | 1:250 |
| Anti-Mouse 555 | Invitrogen | A31570 | 1:250 |
| Anti-Mouse 594 | Invitrogen | 21203 | 1:250 |
| Anti-Mouse 647 | Invitrogen | A31571 | 1:250 |
| Anti-Rabbit 488 | Invitrogen | 21206 | 1:250 |
| Anti-Rabbit 594 | Invitrogen | 21207 | 1:250 |
| Anti-Rabbit 647 | Invitrogen | A-31573 | 1:250 |
| Anti-Rat 488 | Abcam | Ab150153 | 1:250 |
| Anti-Rat 594 | Invitrogen | A-21471 | 1:250 |
| Anti-Rat 647 | Abcam | ab150155 | 1:250 |
| PE- Streptavidin | BioLegend | 405245 | 1:100 |

**Supplementary Table 2: Details of antibodies and lectin.**
